# The influence of IONPs core size on their biocompatibility and activity in in vitro cellular models

**DOI:** 10.1038/s41598-021-01237-y

**Published:** 2021-11-08

**Authors:** Natalia Janik-Olchawa, Agnieszka Drozdz, Damian Ryszawy, Maciej Pudelek, Karolina Planeta, Zuzanna Setkowicz, Maciej Sniegocki, Magdalena Wytrwal-Sarna, Marta Gajewska, Joanna Chwiej

**Affiliations:** 1 AGH University of Science and Technology, Faculty of Physics and Applied Computer Science, Krakow, Poland; 2 Jagiellonian University, Faculty of Biochemistry Biophysics and Biotechnology, Krakow, Poland; 3 Institute of Zoology and Biomedical Research, Jagiellonian University, Krakow, Poland; 4 Nicolaus Copernicus University, Faculty of Medicine, Bydgoszcz, Poland; 5 Academic Centre for Materials and Nanotechnology, AGH University of Science and Technology, Krakow, Poland

**Keywords:** Biological techniques, Biophysics, Cell biology, Health care, Risk factors, Nanoscience and technology

## Abstract

Although the key factor affecting the biocompatibility of IONPs is the core size, there is a lack of regular investigation concerning the impact of the parameter on the toxicity of these nanomaterials. Therefore, such studies were carried out in this paper. Their purpose was to compare the influence of PEG-coated-magnetite NPs with the core of 5, 10 and 30 nm on six carefully selected cell lines. The proliferation rate, viability, metabolic activity, migration activity, ROS levels and cytoskeleton architecture of cells have been evaluated for specified incubation periods. These were 24 and 72-h long incubations with IONPs administered in two doses: 5 and 25 µg Fe/ml. A decrease in viability was observed after exposure to the tested NPs for all the analyzed cell lines. This effect was not connected with core diameter but depended on the exposure time to the nanomaterials. IONPs increased not only the proliferation rate of macrophages—being phagocytic cells—but also, under certain conditions stimulated tumor cell divisions. Most likely, the increase in proliferation rate of macrophages contributed to the changes in the architecture of their cytoskeleton. The growth in the level of ROS in cells had been induced mainly by the smallest NPs. This effect was observed for HEK293T cells and two cancerous lines: U87MG (at both doses tested) and T98G (only for the higher dose). This requires further study concerning both potential toxicity of such IONPs to the kidneys and assessing their therapeutic potential in the treatment of glioblastoma multiforme.

## Introduction

Iron oxide nanoparticles (IONPs) have a privileged position among both magnetic nanomaterials (NMS) and other nanoparticles (NPs) in respect of the possibilities of their use in medicine. The animal experiments and preclinical trials showed that they could be used for cell tracking, drug delivery, tissue repair, contrast enhancement in MRI and cancer treatment using the phenomenon of local hyperthermia^1–8^. What is more, enabling the integration of imaging and therapy, they can be utilized as theranostic agents and contribute to the development of personalized medicine^1,6,8^.

When planning the medical use of nanomaterials based on IONPs, one should consider their physical and chemical properties, which depend on the core material and coating, but above all size which determines, among others, magnetic properties, penetration capacity, and reactivity of NPs.

The magnetite nanoparticles with core diameter below 20 nm become superparamagnetic (SPIONs), which means that they no longer show magnetic interactions after removing the external magnetic field^9–11^. However, they still have much greater magnetic susceptibility than paramagnetic materials^12,13^. From a theranostic point of view, these properties are beneficial as they prevent SPIONs from the agglomeration and aggregation in the suspensions, simultaneously allowing to manipulate them magnetically^14,15^.

Small IONPs can more easily penetrate cells causing damages to their structure and functioning^16,17^. What is more, due to the enhanced surface-to-volume ratio, they may reveal elevated toxicity compared with the larger particles or bulk magnetite^18,19^. In turn, larger IONPs, which do not present superparamagnetic properties, tend to agglomerate due to magnetic attraction. In biological systems, this phenomenon may lead to mechanical injuries and the following cytotoxicity^20^. Thus, the comprehensive studies carried out on cellular in vitro models should be the first step towards evaluation of the size-dependent IONPs biocompatibility and toxicity^19^.

Previous in vitro studies concerning the influence of core size on the biocompatibility of IONPs have been primarily focused on the assessment of the differences in the cytotoxicity of these nanomaterials. For this purpose, the MTT assay^21–26^ or, much less frequently, the trypan blue staining^25,27^ have been used. Despite several published papers including information on changes in the intracellular level of ROS and the actin cytoskeleton after exposure to iron oxide nanoparticles of various sizes^23,27–32^, the knowledge on this subject is still very poor, similarly as the data concerning the effect of core size on the proliferation rate and migration activity of the exposed cells.

Reassuming, according to our best knowledge, there is a lack of regular in vitro studies concerning the influence of core size of IONPs on their toxicity for both normal and cancerous cells. Therefore, the impact of polyethylene glycol (PEG) coated magnetite NPs with three different core diameters (5, 10 and 30 nm) on various cell lines was examined in this paper. The IONPs were administered in two doses 5 and 25 µg Fe/ml, and their influence on the tested cells was measured after 24 and 72-h long exposure. Six cell lines were carefully chosen for the study to determine both possible toxic effects on normal cells and therapeutic potential in the case of cancerous ones. These were human bronchial fibroblasts (NHLF) and human embryonic kidney cells (HEK293T), mouse macrophages (MAC), two human glioblastoma multiforme (GBM) cell lines (U87MG and T98G) as well as GBM cells derived from the patient (KJT23I). To examine the cytotoxicity of tested IONPs on cells, MTT assay was used to assess metabolic activity and trypan blue test to verify viability of cells. Additionally, proliferation rate, migration activity, the level of oxidative stress and the status of cytoskeletal architecture in IONPs-treated and control cells were compared.

## Results

### Characterization of the nanoparticles

The average core diameters of examined IONPs were specified by manufacturers using the transmission electron microscopy (TEM) and assumed as 5, 10 and 30 nm. The supplementary TEM measurements done in our study (Fig. S1 of Supplementary materials) showed the presence in the solutions the particles with spherical cores in the nanometer range and narrow size distributions. The mean core sizes of the examined nanoparticles were 4.63, 13.22 and 28.1 nm what is in quite good agreement with the values presented in the certificates characterizing Sigma-Aldrich nanoparticles 790508, 747319, 747408.

The thermal gravimetric analysis (TGA) carried out in the temperature range 24–500 °C (Fig.S2 of Supplementary materials), confirmed the presence of polymer coating on the NPs core surface which decomposed together with the increase of the temperature, leading to the weight loss of samples. The analysis of TGA curves showed that the total weight loss in the examined temperature range was 49.7, 65.9 and 23.9% for 5, 10 and 30 nm IONPs, respectively. The main progressive stage of the decomposition of the PEG polymer shell on nanoparticles took place in the temperature range from about 200 to about 500 °C and within this range, the weight loss of the samples amounted to 40.1, 59.1 and 19% for 5, 10 and 30 nm NPs.

The results of dynamic light scattering (DLS) measurements for studied nanoparticles are summarized in Table 1. DLS showed systematically larger sizes than TEM what one can see comparing the mean hydrodynamic diameter (d_z_) values with IONPs core sizes. Obviously, the average diameters determined from microscopic visualizations are smaller than those estimated from DLS measurements. Such a result was to be expected as TEM has ability to measure only the magnetite core size of the examined IONPs. What is more, hydrodynamic diameter measured with DLS is heavily weighted toward the largest structures in the solution^37^. Polydispersity index (PDI) values were relatively high for 5 and 10 nm IONPs dispersed in PBS. The dispersion in the culture medium supplemented with 10% vol. fetal bovine serum (FBS) did not affect only the hydrodynamic diameter of 5 nm NPs. In turn, for 10 and 30 nm IONPs the decrease of d_z_ values was found. It probably resulted from the presence of proteins from FBS causing underrepresentation of the real values of hydrodynamic diameter of these NPs. For the largest 30 nm nanoparticles d_z_ decreased almost twice (from 87.4 to 47.0 nm), while PDI increased from 0.18 to 0.51. All the tested IONPs possessed slightly negative ζ-potential values.

**Table 1:** The values of the mean hydrodynamic diameter (d_z_), polydispersity index (PDI), and ζ-potential at 298 K for examined IONPs (c_IONPs_ = 0.01 mg/mL) dispersed in PBS and DMEM with 10% vol. FBS at pH 7.4 (values are the mean ± standard deviation).

Dispersant	Core diameter from manufacturer (nm)	Mean hydrodynamic diameter d_*z*_ (nm)	PDI	ζ-potential (mV)
PBS	5	44.8 ± 0.2	0.41 ± 0.05	−9.8 ± 1.6
	10	45.4 ± 0.2	0.45 ± 0.02	–7.9 ± 1.0
	30	87.4 ± 0.4	0.18 ± 0.01	−4.5 ± 0.8
DMEM/10% vol. FBS	5	43.2 ± 0.8	0.51 ± 0.03	−4.6 ± 2.7
	10	21.0 ± 0.3	0.46 ± 0.04	−1.7 ± 1.6
	30	47.0 ± 0.3	0.51 ± 0.02	−3.4 ± 1.3

### The influence of tested IONPs on proliferation rate of cells

**Figure 1:**
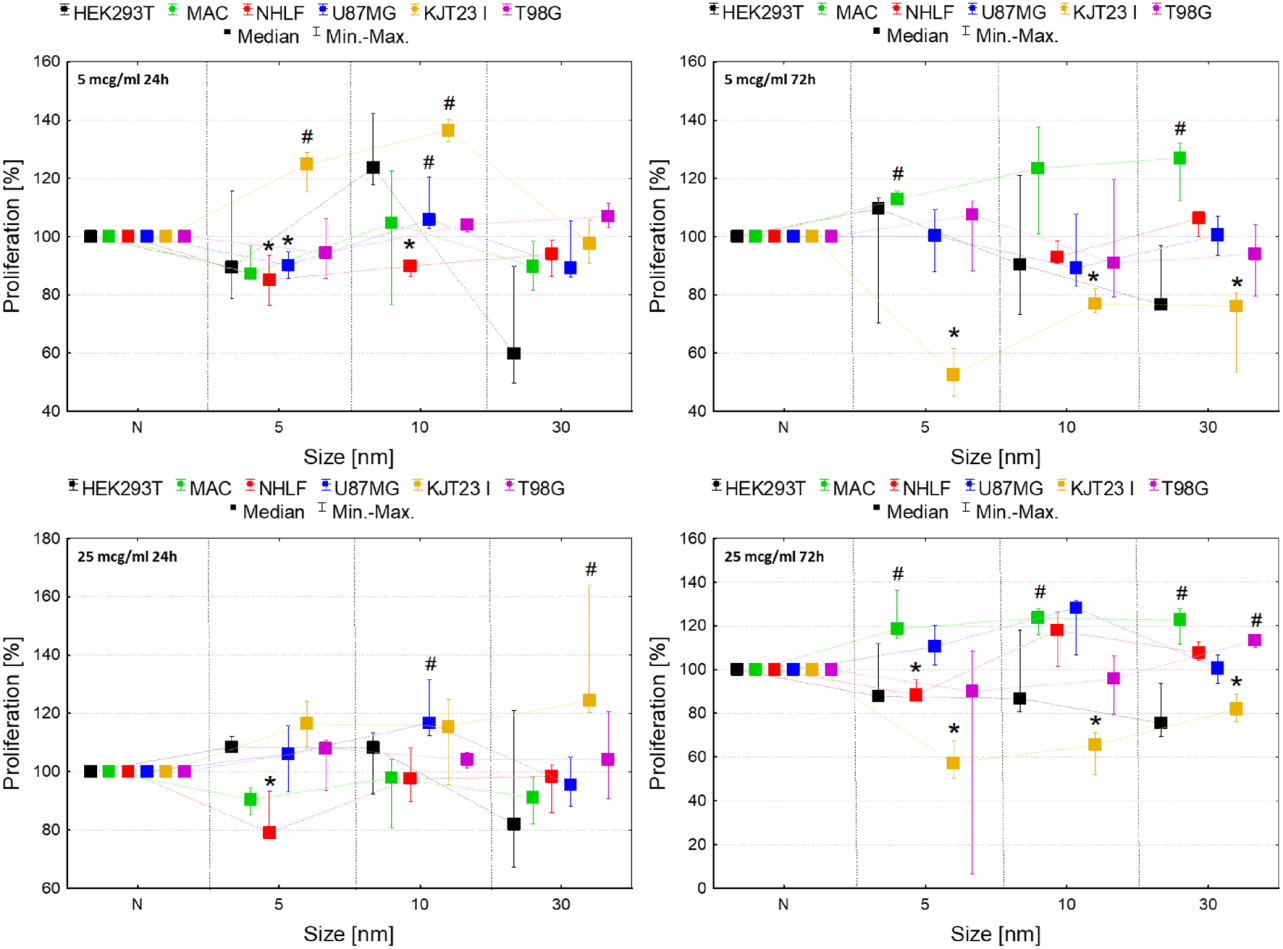
Normalized cell number after 24 and 72-h exposure to IONPs with core diameter of 5 nm, 10 nm and 30 nm in the concentrations of 5 µg Fe/ml and 25 µg Fe/ml comparing to the control group (N). The statistically significant (*p* < 0.05) increases of cell lines proliferation level compared with corresponding N group were marked with “#” while decreases with “*”. The scales were adjusted for each chart so that data presented are the most legible.

The differences in proliferation rate between cells exposed to NPs and control groups (N) are presented in Fig. 1.

As one can see from Fig. 1, for HEK293T cells exposed to IONPs with 5, 10 and 30 nm diameter no statistically significant changes in proliferation were observed. Macrophages manifested significantly (*p* < 0.05) increased proliferation after 72-h exposure to IONPs with sizes of 5 and 30 nm administered in both 5 and 25 µg Fe/ml doses and also 10 nm in 25 µg Fe/ml dose. The proliferation level of NHLF cells was relevantly diminished after 24-h exposure to IONPs with diameter of 5 nm in doses of 5 µg Fe/ml and 25 µg Fe/ml as well as 10 nm in dose of 5 µg Fe/ml. Additionally, the NHLF cells proliferation was lower comparing to control after 72 h from administration of 5 nm IONPs in 25 µg Fe/ml dose.

The proliferation activity of U87MG cell line diminished after 24-h long exposure to 5 nm IONPs in dose of 5 µg Fe/ml. In turn, IONPs 10 nm in size caused statistically relevant increases in proliferation levels after 24 h from their administration in doses of 5 and 25 µg Fe/ml. For KJT23I cell line proliferation activity was significantly increased after 24-h exposure to 5 and 10 nm IONPs in 5 µg Fe/ml dose as well as 30 nm IONPs in dose of 25 µg Fe/ml. However, 72-h long exposure to all examined types of IONPs and both tested doses relevantly diminished the KJT23I cells proliferation. T98G cell line demonstrated only elevated proliferation after 72-h exposure to 30 nm IONPs in higher dose.

### Viability assessment with trypan blue test

**Figure 2:**
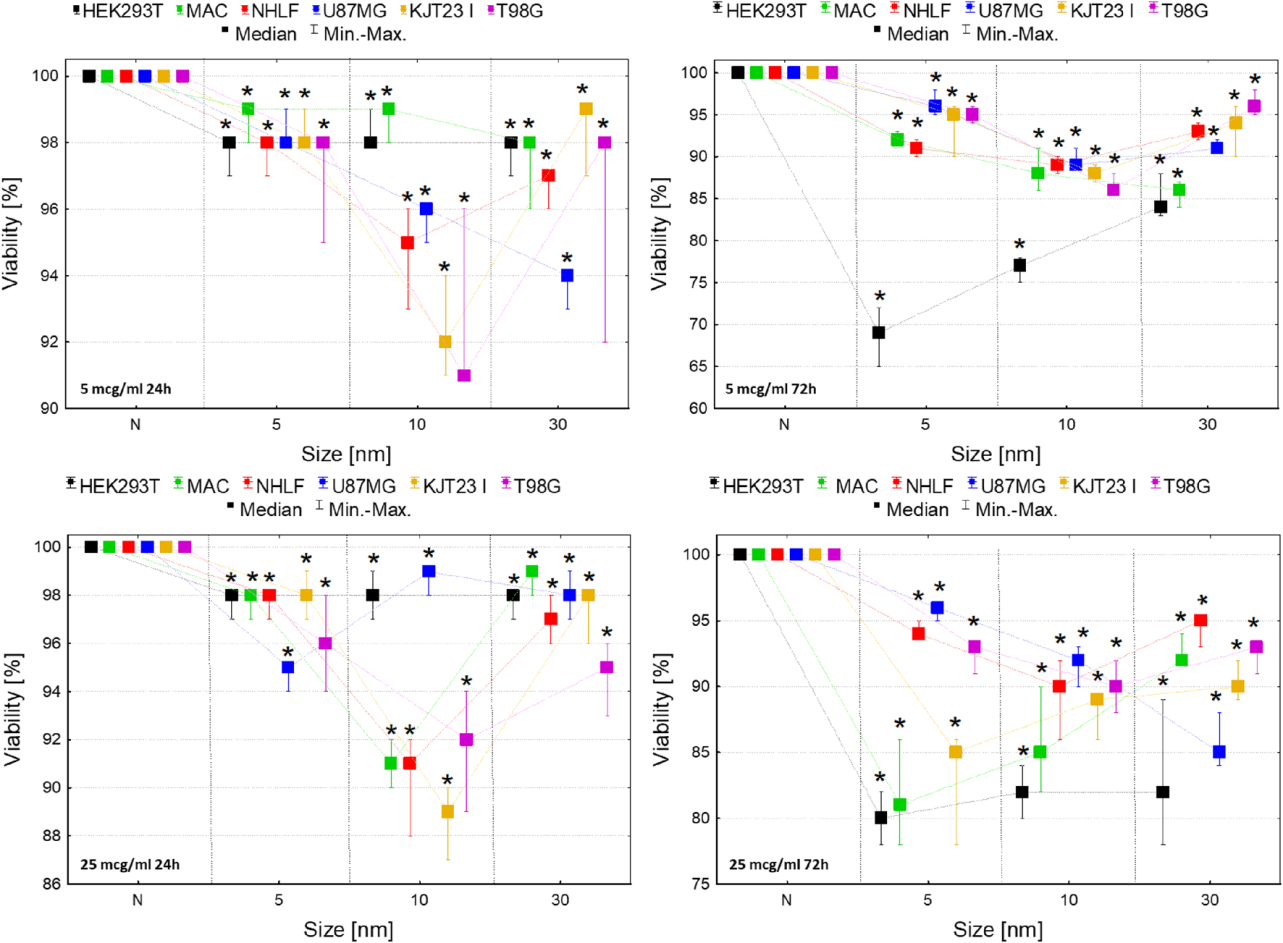
Normalized cell viability after 24 and 72-h exposure to IONPs with core diameter of 5 nm, 10 nm and 30 nm in the concentrations of 5 µg Fe/ml and 25 µg Fe/ml comparing to the control groups (N). The statistically significant (*p* < 0.05) decreases of cells viability compared with corresponding N group were marked with “*”. The scales were adjusted for each chart so that data presented are the most legible.

For the assessment of the influence of IONPs on cell lines viability trypan blue test was applied. Its results, presented in the Fig. 2, demonstrated the statistically significant (*p* < 0.05) decrease in viability of all cells exposed to IONPs in both of the tested doses. However, after 24-h exposure the observed effect seemed to be stronger for KJT23I and T98G lines (8–11% decrease) treated with 10 nm IONPs in both analyzed doses as well as for macrophages and NHLF cells (9% decrease) subjected to the action of these NPs in a higher dose. In case of 72-h exposure, the negative impact on cell viability was the most pronounced for HEK293T (15–30%) cell line treated with all tested nanoparticles in both doses. Additionally, after 72 h relatively strong decrease in viability (10–19%) was observed for macrophages and KJT23I cells exposed to 5 and 10 nm IONPs in 25 µg Fe/ml dose.

### Metabolic activity assessment with MTT assay

**Figure 3:**
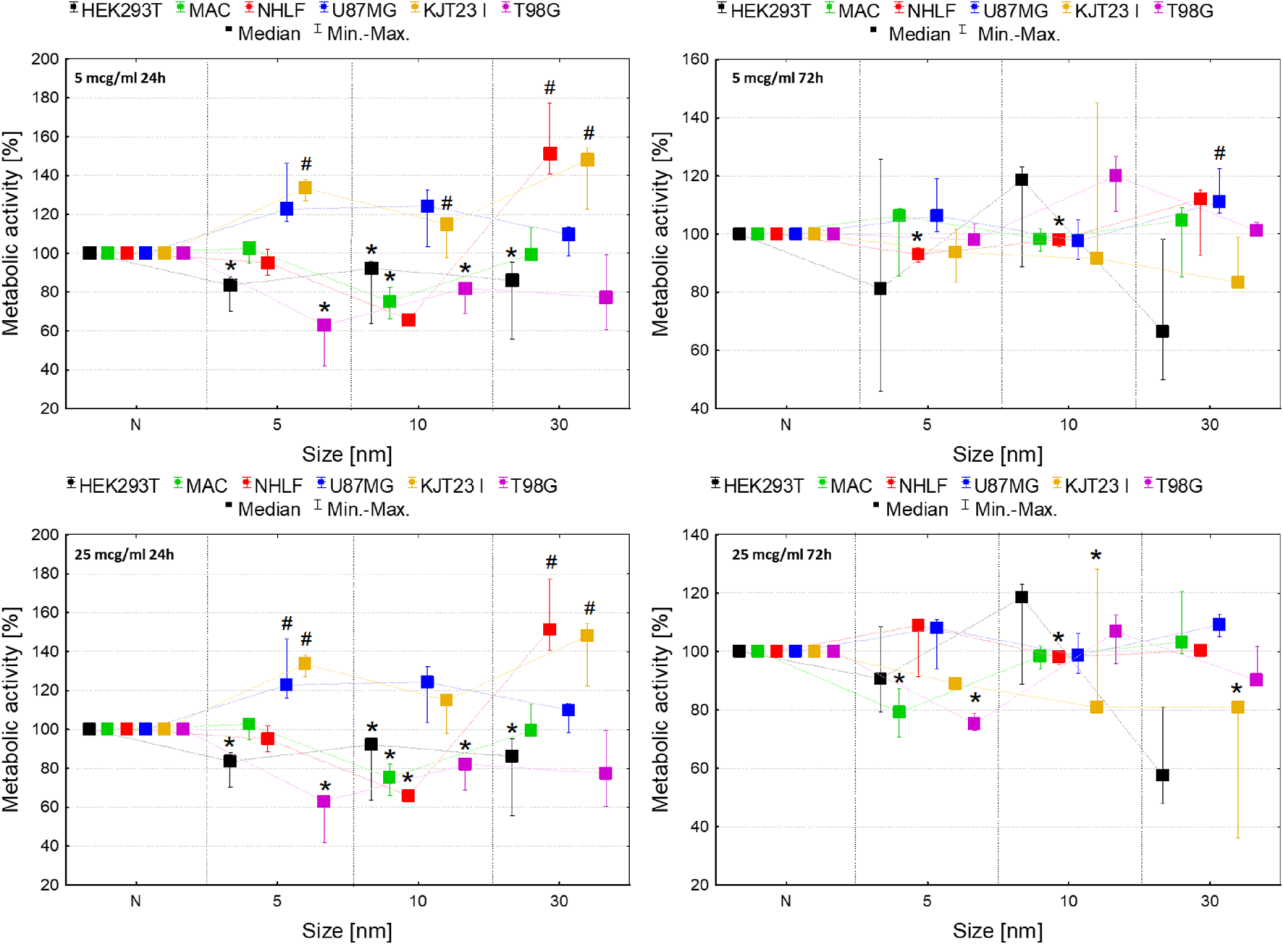
Normalized cell metabolic activity after 24 and 72-h exposure to IONPs with the core diameter of 5 nm, 10 nm and 30 nm in the concentrations of 5 µg Fe/ml and 25 µg Fe/ml comparing to the control groups (N). The statistically significant (*p* < 0.05) increases of cell lines metabolic activity compared with corresponding N group were marked with “#” while decreases with “*”. The scales were adjusted for each chart so that data presented are the most legible.

According to Fig. 3, the metabolic activity of HEK293T cell line decreased significantly (*p* < 0.05) after 24 h of exposure to all analyzed IONPs in both 5 and 25 µg Fe/ml doses. Macrophages revealed diminished level of this parameter after 24 h from administration of 10 nm IONPs in doses of 5 and 25 µg Fe/ml as well as after 72-h treatment with 5 nm IONPs in a higher dose.

Increased metabolic activity was observed for NHLF cells after 24-h exposure to 30 nm IONPs in 5 and 25 µg Fe/ml doses. In turn, 24-h exposure of this cell line to 10 nm IONPs in dose of 25 µg Fe/ml and 72-h exposure to the IONPs in 5 and 25 µg Fe/ml doses decreased the value of the analyzed parameter. The similar relation was found for NHLF cells treated with 5 nm IONPs in 5 µg Fe/ml dose for 72 h. The metabolic activity of U87MG cells was significantly elevated after 72-h exposure to 30 nm IONPs in dose of 5 µg Fe/ml as well as in 24th hour from administration of 5 nm IONPs in 25 µg Fe/ml dose. KJT23I cells showed an elevated level of the parameter after 24-h exposure to 5 and 30 nm IONPs in dose of 5 and 25 µg Fe/ml as well as 10 nm IONPs in 5 µg Fe/ml dose. In turn, after 72 h from administration of 10 and 30 nm IONPs in dose of 25 µg Fe/ml, metabolic activity of this cell line significantly decreased. The metabolic activity of T98G cells diminished significantly after 24-h long exposure to 5 and 10 nm IONPs in both analyzed doses and also after 72 h from administration of 5 nm IONPs in a higher dose.

### The influence of tested IONPs on cell motility

**Figure 4:**
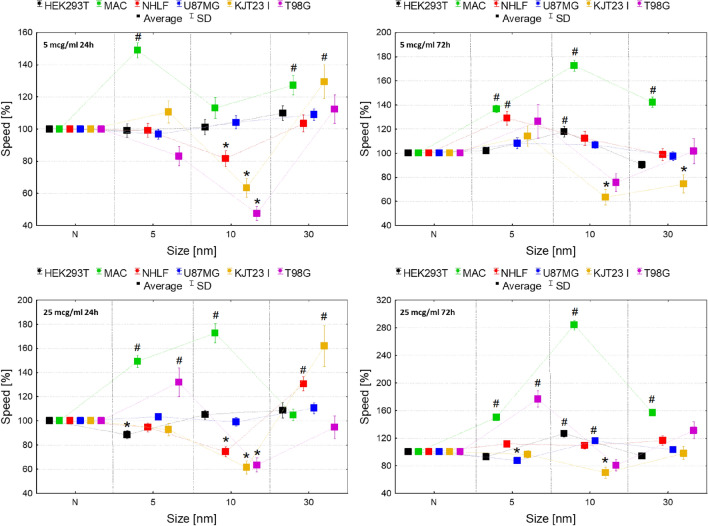
Normalized cell speed after 24 and 72-h exposure to IONPs with the diameter of 5 nm, 10 nm and 30 nm in the concentrations of 5 µg Fe/ml and 25 µg Fe/ml comparing to the control group (N). The statistically significant (*p* < 0.05) increases of cell lines motility compared with corresponding N group were marked with “#” while decreases with “*”. The scales were adjusted for each chart so that data presented are the most legible.

**Figure 5:**
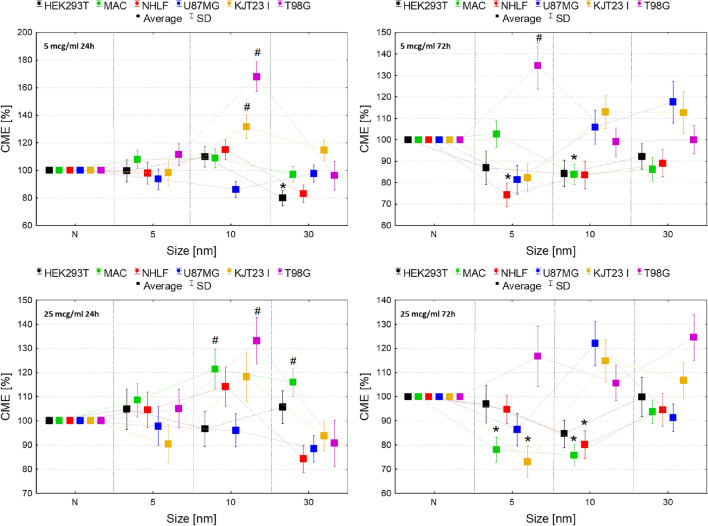
Normalized CME coefficient values after 24 and 72-h exposure to IONPs with the diameter of 5 nm, 10 nm and 30 nm in the concentrations of 5 µg Fe/ml and 25 µg Fe/ml comparing to the control group (N). The statistically significant (*p* < 0.05) increases of CME compared with corresponding N group were marked with “#” while decreases with “*”. The scales were adjusted for each chart so that data presented are the most legible.

The assessment of the influence of IONPs on cell motility was based on two parameters. The first one was cells speed showed in the Fig. 4 and the second one was coefficient of movement efficiency (CME) presented in the Fig. 5. The CME is calculated as the ratio of cell displacement to the length of its trajectory and it gives information on how particular IONPs and administered doses modify the character of cellular movement. The dot-plots depicting the displacement and the total length of trajectory (distance traveled) for single cells and the circular plots presenting trajectories of individual cells are additionally placed in the Supplementary Information (Figures S3-S8).

As one can see from Fig. 4, the HEK293T cells revealed significantly (*p* < 0.05) enhanced motility after 72-h exposure to 10 nm IONPs in both 5 and 25 µg Fe/ml dose. In turn 24-h treatment with 5 nm IONPs in dose of 25 µg Fe/ml diminished the movement speed of this cell line. Macrophages, in general, showed increased motility after 24 and 72-h exposure to 5 nm IONPs at both of doses tested. The speed of the cells treated with 10 nm IONPs was elevated after 24 h from their administration in 25 µg Fe/ml dose and after 72 h from treatment with dose of 5 and 25 µg Fe/ml. IONPs with the core diameter of 30 nm also enhanced macrophages motility. This effect was observed after 24-h exposure to the lower dose as well as after 72 h from NPs administration in 5 and 25 µg Fe/ml doses. The speed of NHLF cells was significantly diminished after 24-h treatment with 10 nm IONPs in both of the analyzed doses. In turn, after 24 h from administration of 30 nm IONPs in dose of 25 µg Fe/ml and 72-h long exposure of cells to 5 nm IONPs in 5 µg Fe/ml dose, their motility was significantly elevated. U87MG cells speed was significantly diminished after 72 h of treatment with 5 nm IONPs in 25 µg Fe/ml dose while it raised after exposure to 10 nm in the same dose. The motility of KJT23I cells decreased significantly after 24 and 72 h from administration of 10 nm IONPs in both tested doses. Treatment of the cell line with 30 nm NPs in dose of 5 and 25 µg Fe/ml caused an increase of their speed after 24 h. In turn, 72-h exposure to 30 nm IONPs in 5 µg Fe/ml dose reduced speed of KJT23I cells. The T98G cells showed a significant decrease in motility after 24-h treatment with 10 nm IONPs in both tested doses. However, after 24 and 72-h long exposure to 5 nm IONPs in the 25 µg Fe/ml dose, their speed was elevated.

According to the Fig. 5, the movement efficiency of HEK293T cells was significantly (*p* < 0.05) diminished after 24-h exposure to 30 nm IONPs in dose of 5 µg Fe/ml. Macrophages showed reduced CME after 72 h from administration of 10 nm IONPs in 5 and 25 µg Fe/ml dose and after 72 h from treatment with 5 nm IONPs in higher dose. In the case of NHLF cells movement efficiency decreased after 72 h from administration of 5 nm IONPs in dose of 5 µg Fe/ml as well as 10 nm IONPs in 25 µg Fe/ml dose. No statistically relevant changes in value of CME were found for U87MG cells. KJT23I cells revealed enhanced motility after 24-h exposure to 10 nm IONPs in 5 µg Fe/ml dose. However, the CME value for the cell line diminished significantly after 72 h from administration of 5 nm IONPs in higher of the tested dose. The movement efficiency of T98G cells increased significantly after 24-h treatment with 10 nm IONPs in both 5 and 25 µg Fe/ml dose and after 72-h long exposure to 5 nm IONPs in a dose of 5 µg Fe/ml.

### The influence of tested IONPs on ROS production

**Figure 6:**
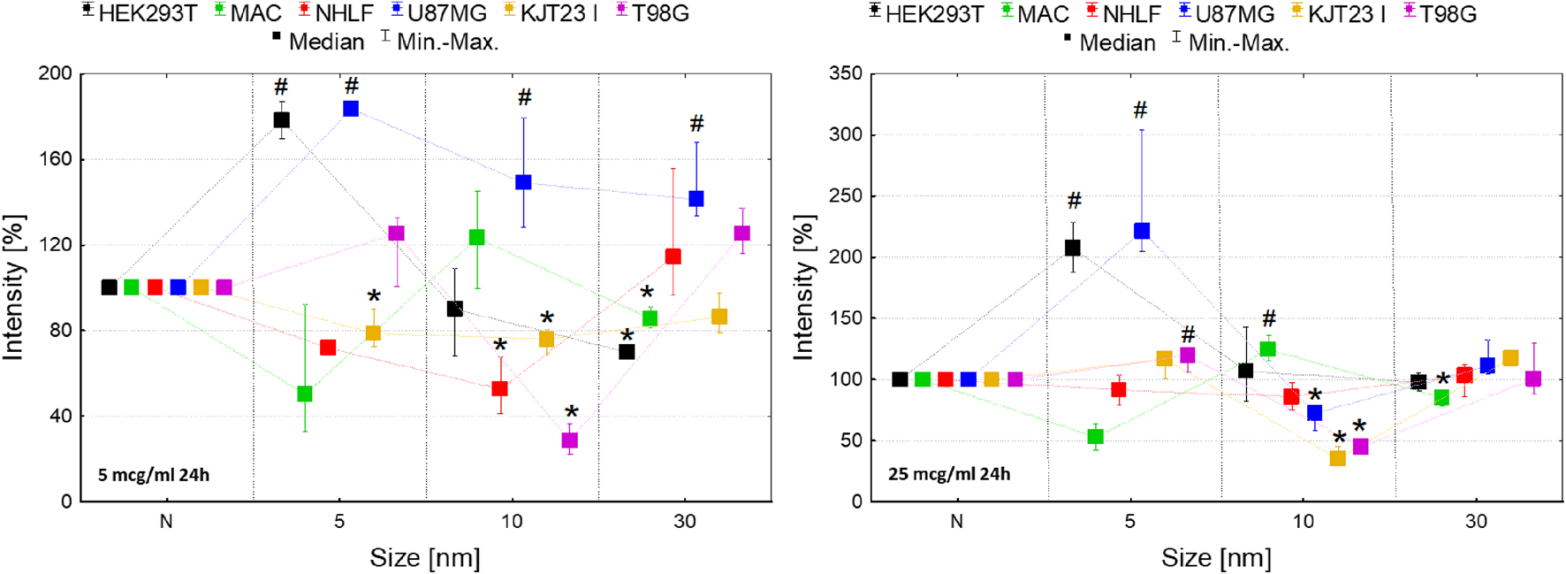
ROS levels after 24 h of exposure to IONPs having the diameters of 5 nm, 10 nm and 30 nm in the concentrations of 5 µg Fe/ml and 25 µg Fe/ml comparing to the control group (N). The statistically significant (*p* < 0.05) increases in ROS level compared with corresponding N group were marked with “#” while decreases with “*”.

**Figure 7:**
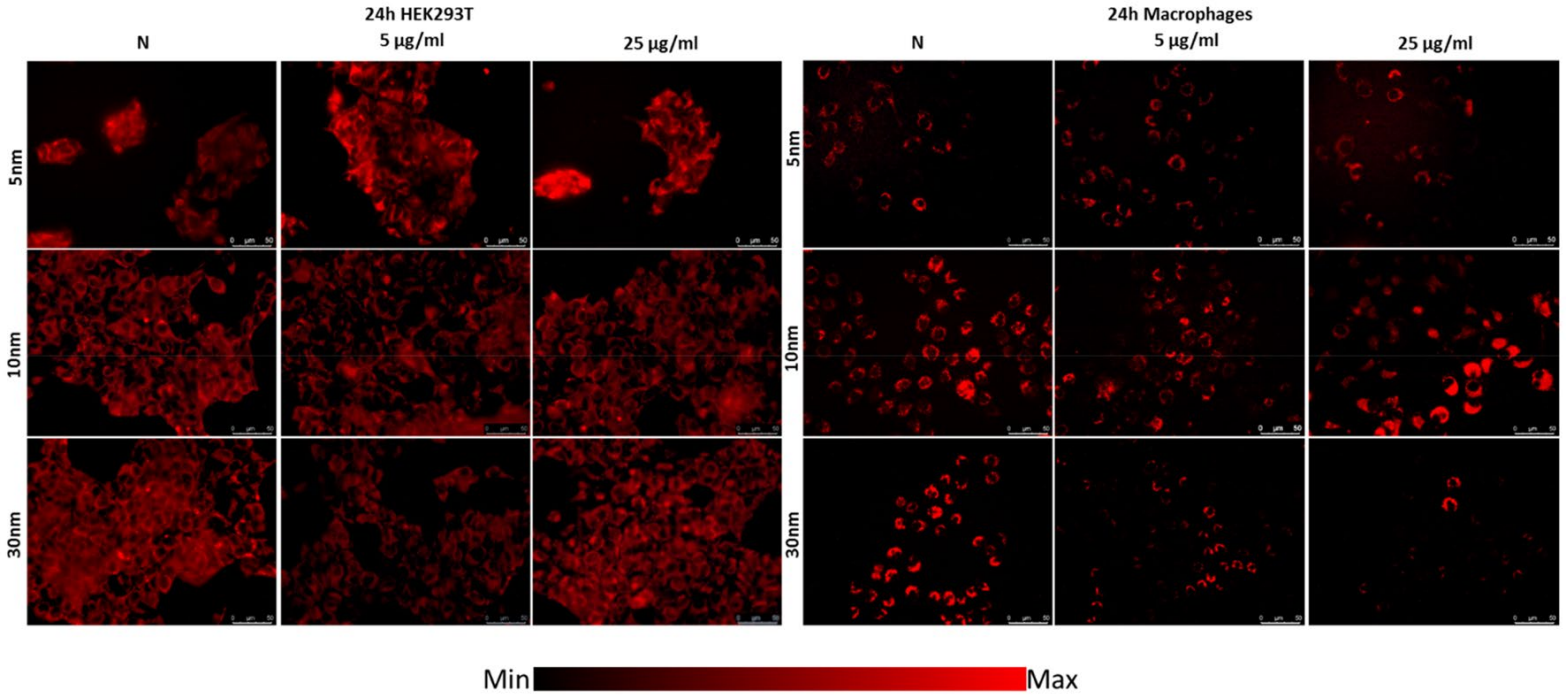
Microscopic images of HEK293T cells and macrophages presenting ROS levels after 24 h of exposure to IONPs with 5 nm, 10 nm and 30 nm diameters in concentrations of 5 µg Fe/ml and 25 µg Fe/ml compared with control group (N).

**Figure 8:**
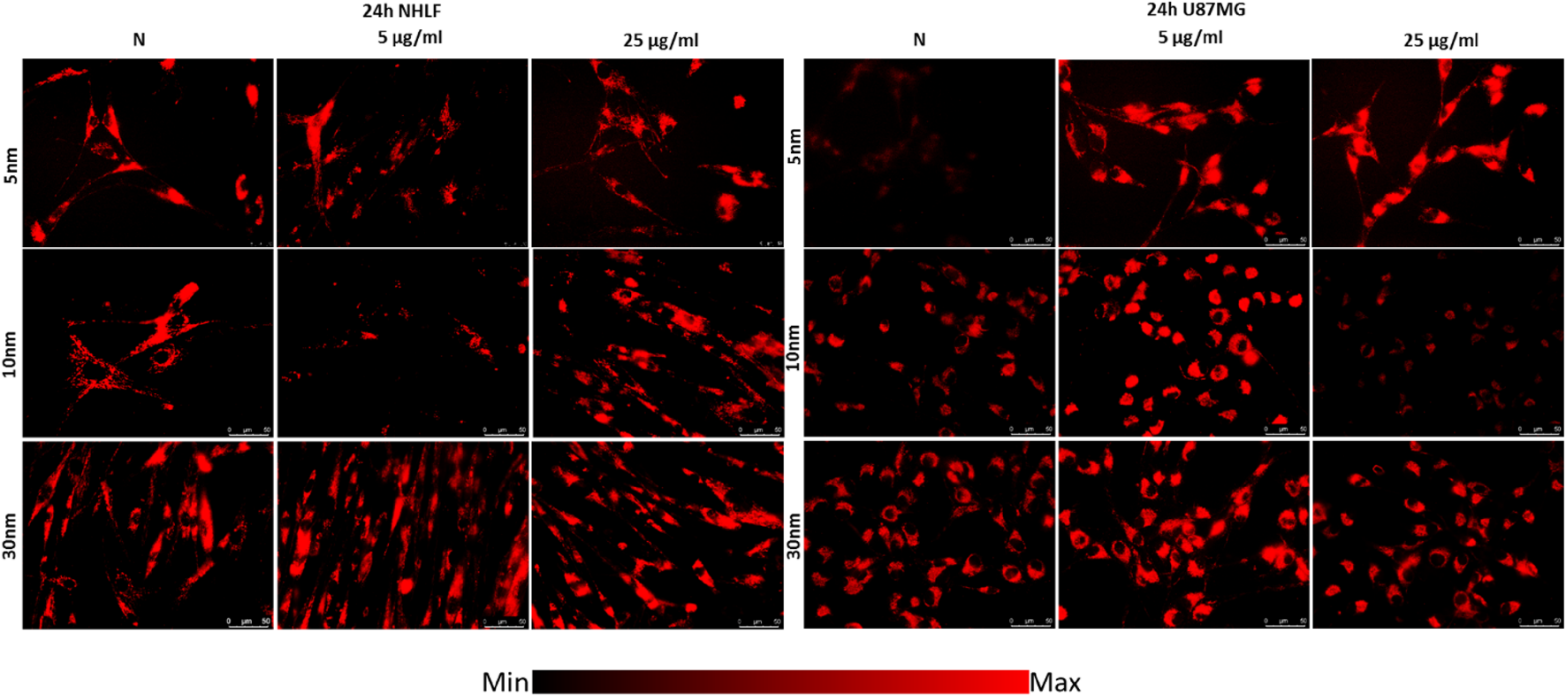
Microscopic images of NHLF and U87MG cells presenting ROS levels after 24 h of exposure to IONPs with 5 nm, 10 nm and 30 nm diameters in concentrations of 5 µg Fe/ml and 25 µg Fe/ml compared with control group (N).

**Figure 9:**
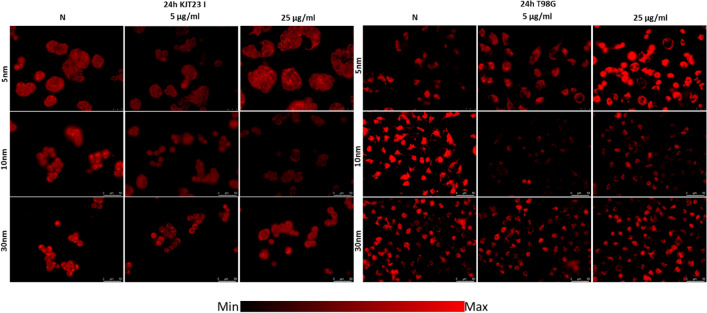
Microscopic images of KJT23I and T98G cells presenting ROS levels after 24 h of exposure to IONPs with 5 nm, 10 nm and 30 nm diameters in concentrations of 5 µg Fe/ml and 25 µg Fe/ml compared with control group (N).

The influence of analyzed IONPs on ROS production in cells was evaluated quantitatively (Fig. 6) and qualitatively (Figs. 7, 8, 9) based on intracellular ROS probe fluorescence after 24-h exposure. The results obtained for cell lines exposed to NPs action were compared to those for corresponding norms to assess ROS activity changes after IONPs administration.

As shown in Fig. 6, a significant (*p* < 0.05) increase of ROS production in HEK293T cells exposed to 5 nm IONPs in both analyzed doses was found. The opposite relation for this cell line was noticed after the treatment with 30 nm IONPs in dose of 5 µg Fe/ml. Macrophages revealed diminished ROS production after the treatment with 30 nm NPs in both tested doses. However, the exposure of the cell line to 10 nm IONPs in the 25 µg Fe/ml dose resulted in the enhancement of ROS level. The anomalies in ROS production for HEK293T cell line and macrophages are visible in fluorescent images presented in the Fig. 7.

The ROS activity of NHLF cells diminished significantly after 24 h from administration of 10 nm IONPs in dose of 5 µg Fe/ml. U87MG cells showed elevated ROS level when exposed to 5, 10 and 30 nm IONPs in the lower dose as well as after the treatment with 5 nm IONPs in the dose of 25 µg Fe/ml. Whereas after administration of 10 nm IONPs in a higher dose, ROS activity of this cell line decreased. The fluorescent images obtained for NHLF and U87MG cell lines are presented in the Fig. 8.

The ROS production in KJT23I cells was significantly diminished after exposure to 5 nm IONPs in dose of 5 µg Fe/ml as well as 10 nm IONPs in both examined doses. T98G cells also demonstrated relevant decrease in ROS level after treatment with 10 nm IONPs in both doses but after administration of 5 nm IONPs in the higher dose, the ROS activity for this cell line was elevated. The changes in ROS levels in KJT23I and T98G cell lines are presented in Fig. 9.

### Analysis of changes in cytoskeleton architecture

**Figure 10:**
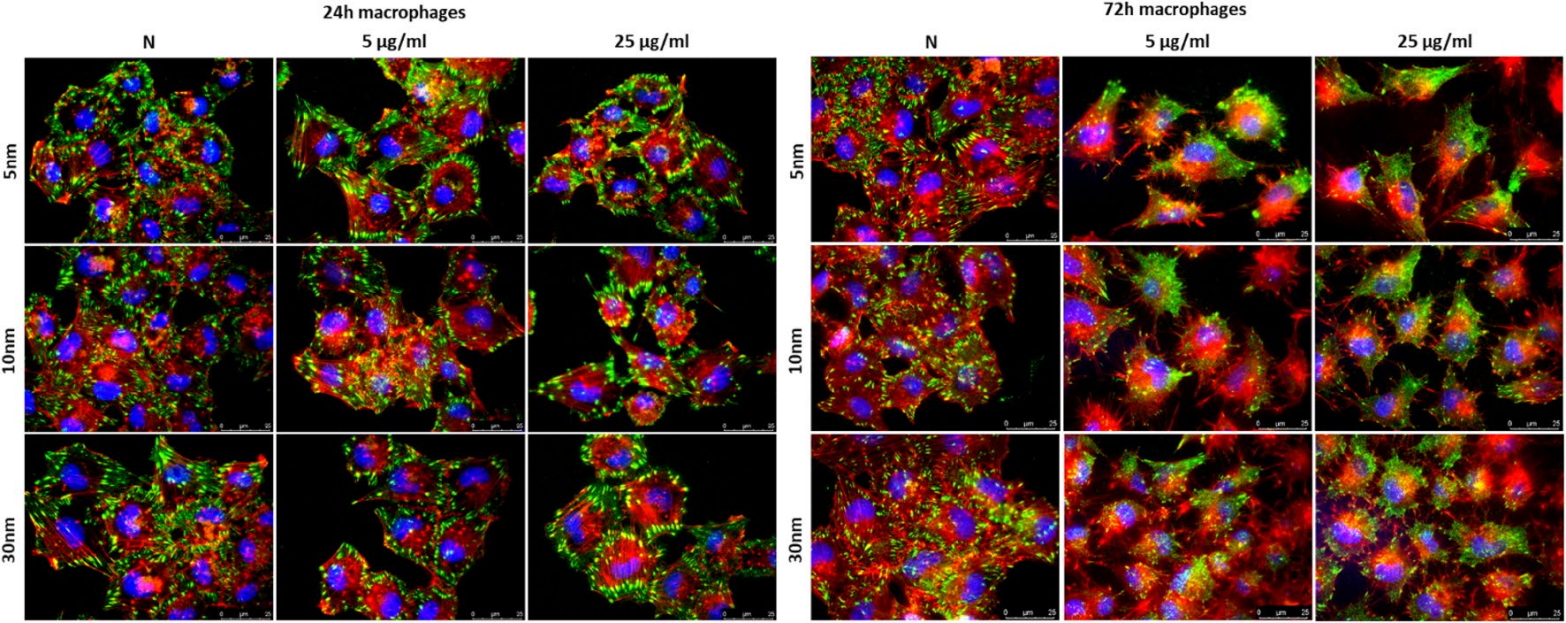
Cytoskeleton architecture of macrophages after 24 and 72-h long of exposure to IONPs with 5 nm, 10 nm and 30 nm core diameters in concentrations of 5 µg Fe/ml and 25 µg Fe/ml compared with control groups (N). The F-actin filaments are stained on red colour, vinculin on green and DNA on blue.

**Figure 11:**
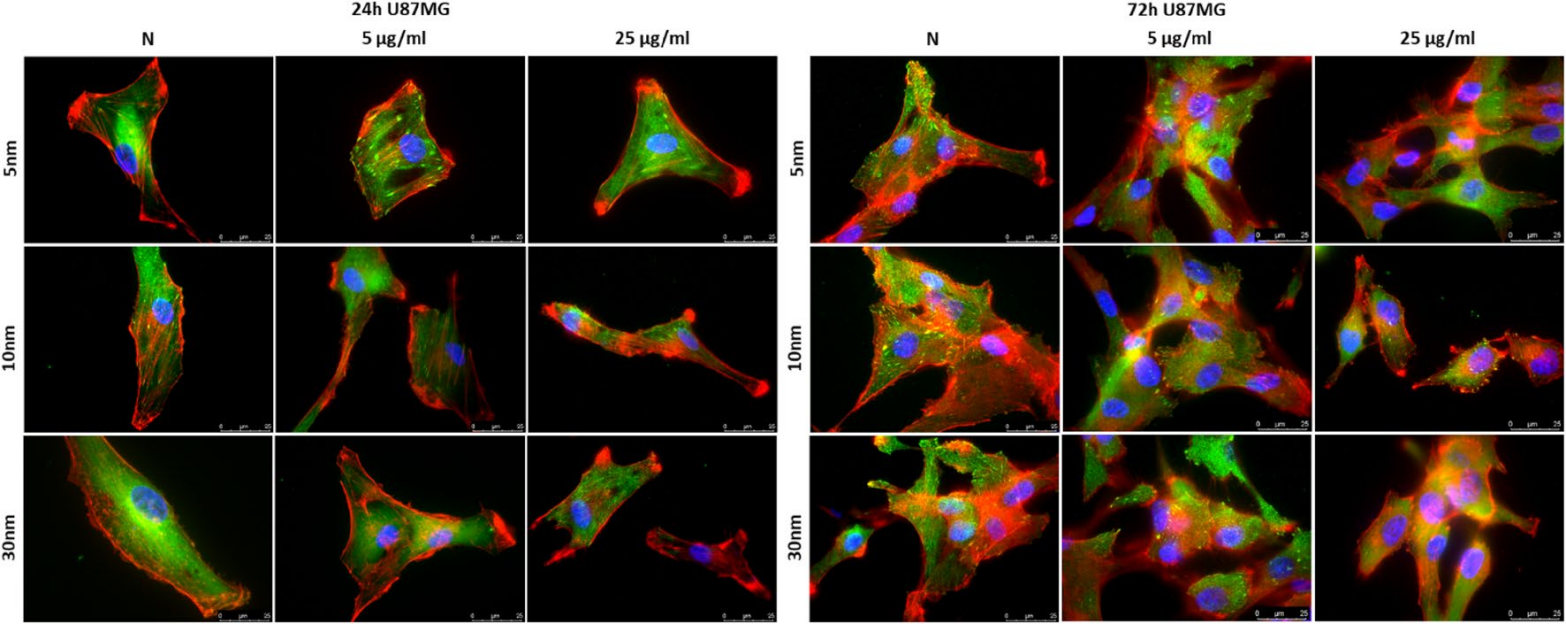
Cytoskeleton architecture of U87MG cells after 24 and 72-h long of exposure to IONPs with 5 nm, 10 nm and 30 nm core diameters in concentrations of 5 µg Fe/ml and 25 µg Fe/ml compared with control groups (N). The F-actin filaments are stained on red colour, vinculin on green and DNA on blue.

The results obtained using a fluorescence microscopy with TIRF module demonstrated changes in cytoskeleton organization and cell morphology for macrophages and U87MG line after the treatment with IONPs. For macrophages (Fig. 10) the observed effect seemed the strongest after 72-h long exposure to all IONPs in the dose of both 5 and 25 µg Fe/ml. The polarization of the cell body together with a decrease in the size and number of focal contacts were noticed. This phenomenon was not observed in the culture of untreated cells showing a more complex focal contacts architecture and relatively pronounced stress fibers (F-actin). For cancerous cell lines, the influence of IONPs on the architecture of selected elements of the cytoskeleton was not as evident. Nevertheless, U87MG cells exposed to the higher dose of IONPs with core sizes of 10 and 30 nm for both 24 and 72-h seem to present reduction in the number of focal contacts and stress fibers (Fig. 11).

The remaining cell lines showed no response to the tested NPs in all concentration ranges in the context of architecture of the cytoskeleton rearrangements what one can see in the Figures S9-S12 of Supplementary materials presenting the results of immunofluorescence staining of F-actin and vinculin obtained for NHLF, HEK293T, T98G and KJT23I cells.

## Discussion

The results of in vitro studies cannot be usually directly translated into in vivo models which are pivotal and mandatory before the first-in-human trials. However, they play a very important role at the first stage of any biomedical research including those in the field of nanomedicine^1–3^ . The in vitro experiments provide the mechanistic information on the toxicity of nanoparticles, in particular their genotoxicity, cytotoxicity, the possibility of oxidative stress induction or the development of inflammatory processes in cells and therefore they are a very important step in the complex process of the enhancement of IONPs biocompatibility and biomedical potential^4^.

Our results clearly show that IONPs influence on proliferation strongly depends on the tested cell line. HEK293T cells did not demonstrate changes in proliferation level after exposition to any of the examined NPs. In turn, the rate of NHLF and U87MG cells proliferation was modified only by small IONPs with 5 and 10 nm cores. In case of the first of the mentioned cell lines, NPs reduced the proliferation rate. For U87MG cells decreased rate of cell divisions was noticed for 5 nm while increased for 10 nm IONPs. For two of examined cell lines the time-dependent influence of IONPs with all sizes on proliferation was observed. KJT23I glioblastoma cells showed an enhanced proliferation rate after 24-h long exposure to IONPs and a decreased cell divisions after 72 h from NPs administration. In turn, T98G GBM cell line revealed elevated proliferation level only after prolonged exposure to 30 nm IONPs in higher dose. For macrophages, the level of proliferation increased after 72-h long treatment with all tested NPs.

There are no such comprehensive studies concerning the effect of NPs size on the proliferation rate of cells so far. Abakumov et al*.* examined the toxicity of IONPs with hydrodynamic sizes comparable with ones used in the current study and utilized for this purpose the human fibroblasts and GBM cell line U251. The authors did not found any changes in confluence of glioma cells treated with IONPs^23^. However, it should be noticed that confluence is not an appropriate proliferation determinant, especially when cells differ with regard to adhesion. In the present study, enhanced proliferation rate was observed for U87MG cells after the exposure to 10 nm IONPs as well as T98G cell line treated with 30 nm NPs. Such an effect for cancerous cell lines is especially undesirable and may indicate the IONPs ability to enhance the rate of tumor development^38^.

Regardless of the core size, the magnetite nanoparticles caused a decrease in the viability of the tested cell lines measured with the trypan blue test. The observed effect seemed to be independent on the dose of nanoparticles, but increased with the time of exposure to the examined nanomaterials. Given the defined size of the particles and the applied dose, the viability of the cells was lower after 72 than 24-h for all the tested cell lines. At the same time, this effect was the most pronounced for the HEK293T line and macrophages. After 24-h long exposure to NPs, the strongest negative influence on viability was found, at both tested doses for IONPs with 10 nm core. Longer exposure time, especially to larger NPs doses, was connected with the highest decrease of viability for 5 nm IONPs.

The MTT assay is one of the most commonly used colorimetric tests to evaluate cell viability and detection of nanomaterials toxicity. It is based on the conversion of 3-[4,5-dimethylthiazol-2-yl]-2,5-diphenyltetrazolium bromide (MTT) to formazan by mitochondrial dehydrogenases^39^. The results of carried MTT tests showed that magnetite nanoparticles influenced the metabolic activity of all examined cell lines and their effect on cells was the most pronounced after 24-h long exposure.

Metabolic activity of HEK293T and KJT23I cells was influenced by treatment with all tested NPs. However, in the case of kidney cells, metabolic activity always decreased whilst for patient-derived GBM cells the parameter was increasing after 24-h long exposure and after the next 48-h it was lower or did not differ from the values obtained for controls. Increased metabolic activity of cancer cells may be connected with numerous mutations of their genes, including EGFR and p53, that may lead to increased cell viability in the condition of the exposure to IONPs^23,40^. IONPs with smaller core diameters (5 nm and 10 nm) more often led to decrease in metabolic activity of the studied cells. This may result from a few facts. The smaller IONPs after the cellular uptake degrade faster compared to their larger analogues. They are also easier phagocytized and may accumulate in the cell at higher concentration. Additionally, smaller IONPs have larger active surface what causes that they are more effective in production of reactive oxygen species than the larger NPs^41,42^.

The effect of IONPs core size on cell metabolic activity was observed for bronchial fibroblasts. NPs with core diameters of 5 and 10 nm either had no impact on the metabolic rate of NHLF cells or led to its decrease. In turn, larger IONPs increased the level of metabolic activity of this cell line. The basic functions performed by bronchial fibroblasts are the production of elastin, collagen III and extracellular matrix proteoglycans. These cells take part in the processes of tissue repair and remodeling. Although the accumulation of fibroblasts in places of inflammation is usually associated with tissue repair after injury, they excessive proliferation may also contribute to the development of disease states^43,44^. There are reports that the exposure to nanoparticles may induce an inflammatory reaction^45^. In this context, the increased metabolic activity of bronchial fibroblasts after exposure to IONPs with 30 nm magnetite core may suggest mobilization of cells in the answer of NPs induced inflammation process.

The migration assay involving the measurements of cell speed, distance and CME was not used so far in the regular studies of core size-dependent cytotoxicity of IONPs. Wu et al*.* examined the influence of IONPs with different core sizes (31 nm and 38 nm) on HUVEC cell line. Using a modified Boyden chamber, they observed reduced migration of the cells exposed to tested IONPs^46^. In the light of our findings, the strongest IONPs influence on motility was observed for macrophages which speed increased after the exposure to all of tested NPs sizes. The motility of HEK293T, U87MG and T98G cells changed only due to the administration of small-sized IONPs (5 nm and 10 nm). Higher doses of 5 nm NPs contributed to decrease in the speed of HEK293T and U87MG cells. However, the analogous concentration of 10 nm IONPs increased this parameter for both of the aforementioned cell lines. The opposite relationship was observed for T98G cells. In the case of NHLF cells, 5 and 30 nm nanoparticles contributed to increase in their motility, while IONPs of intermediate size diminished the speed of lung fibroblasts. No influence of the smallest nanoparticles on GBM cell line KJT23I speed was observed. 10 nm IONPs, regardless of the incubation time, reduced of these cells speed. In turn, the effect of the largest NPs on KJT23TI cells depended on the incubation time—cell speed increased after 24- and decreased after 72-h long exposure. The elevated speed of movement observed for macrophages exposed to IONPs and, under some conditions also for other cells, did not translate into an increase in the movement efficiency measured by the CME parameter. Moreover, in some cases, the motility decrease after exposure to the tested NPs was accompanied by an enhanced CME (T98G, KJT23TI cell lines after exposure to 10 nm IONP). In this context, it should be emphasized that observed increase in motility and movement efficiency of GBM cell lines after exposure to some of the tested NPs may indicate their ability to enhance the metastatic potential of cancerous cells^47–50^.

In light of our results, the increase in the level of reactive oxygen species in cells is primarily induced by nanoparticles with the smallest core diameter (5 nm). This effect was observed for HEK293T cells and two cancerous lines: U87MG (at both doses tested) and T98G (only for the higher dose). Larger nanoparticles much more often show the opposite effect on the tested cells, contributing to reducing of their ROS levels. The exceptions are U87MG cells for which the ROS level increases after exposure to a lower dose of all tested nanoparticles.

According to the research conducted so far, ROS formation in cells exposed to IONPs is usually significantly higher than for those subjected to microparticles of magnetite^27,51^. However, these results cannot be extrapolated to magnetite NPs with different core diameters. Although smaller NPs diameter is connected with the larger surface area/volume ratio and the same more surface interactions, this does not always translate into greater efficiency of smaller nanoparticles regarding intracellular production of ROS^29^.

Elevated ROS levels observed in cells after the exposure to IONPs may be associated with the exhibited by magnetite NPs catalytic activity analogous to peroxidase^52^. Magnetite mimics the action of the enzyme through the well-known Fenton’s and Haber–Weiss reactions, which convert endogenous H_2_O_2_ into a highly cytotoxic hydroxyl radical that can induce cancer cell death^53–55^. This mechanism states the base of the newly defined chemodynamic therapy connected with increased production of free radicals and should be further studied, especially in case of U87G cell line, for which decreased proliferation rate was also observed after the exposure of lower dose of 5 nm IONPs.

The actin cytoskeleton directly supports cell migration, adhesion, shape change as well as endo- and exocytosis^56^. The carried immunofluorescence stainings showed disturbances in the cytoskeleton architecture as well as changes in morphology of some cell lines after the treatment with the tested nanoparticles. The most obvious cytoskeleton changes were noted for macrophages—phagocytic cells—after 72-h long incubation with all examined IONPs in both doses. However, initial signs of F-actin/vinculin remodelling were observed for macrophages also for 24-h exposure to NPs. The progress of the phagocytosis process needs remodelling of the cell architecture in which, according to the existing literature data, the actin cytoskeleton participates^57,58^. Moreover, NPs treatment is often related to the interference with intracellular redox homeostasis which may also influence cytoskeleton remodeling process^32,57^. However, we did not found for macrophages the correlation between the cytoskeleton remodeling and elevated mitochondrial ROS level measured after 24-h incubation with examined NPs. The observed polarization of the cell body and decrease in the size and number of focal contacts seem rather be connected with the increased proliferation rate noticed for the 72-h long exposure to IONPs.

## Conclusion

IONPs size is the key factor defining their magnetic properties, toxicological profile as well as biocompatibility. What is more, even NPs with the same size may elicit different biological responses depending on the exposed system. Results presented in the frame of our study clearly show that observed changes in cell life parameters, including metabolic activity, proliferation, migration, ROS production, and cytoskeleton architecture, are related not only to the tested IONPs and exposure time but also to the analyzed cell line types. Due to the currently observed increase in the risk of intended and accidental exposure to IONPs, the knowledge of adverse effects and following health hazards associated with it is extremely important. Therefore, in our opinion, the presented regular study concerning the size-dependent influence of IONPs on various cellular in vitro models is of great value for environmental science development especially in the field of biological systems.

## Methods

### Examined IONPs

IONPs having the magnetite core with diameters of 5, 10 and 30 nm, coated with polyethylene glycol (PEG) and dispersed in distilled water in concentration of 1 mg/ml were purchased from Sigma-Aldrich (790508, 747319, 747408). Initial stock solution concentration of Fe in the form of IONPs was precisely defined by the total reflection X-ray fluorescence (TXRF) method using Rigaku Nanohunter II spectrometer. For the experiment purposes, the starting solution was diluted in PBS or cell culture medium to a final Fe concentration of 5 and 25 µg/ml.

The additional transmission electron microscopy (TEM) measurements were done to determine the shape and the distribution of the core diameters for studied IONPs. TEM investigations were done in the Academic Centre of Materials and Nanotechnology on a Tecnai TF20 X-TWIN (FEG) microscope (Thermo Scientific) working at an accelerating voltage of 200 kV. The solutions of NPs used in the study were drop casted on the carbon coated copper grids. The performed core size analysis was based on a series of bright field (BF) high resolution images. At least 100 particles were measured manually for each sample and the mean sizes of particles cores were calculated by fitting a standard lognormal model to the size distribution data.

To confirm the presence of polymer coating on the magnetite cores of IONPs the thermal gravimetric analysis (TGA) was done using a Simultaneous Thermal Analyzers SDT Q600 (TA Instruments, New Castle, DE, USA). The samples subjected to prior lyophilization were measured in a powder form and heated up to 500 °C with a heating rate of β  = 10 °C/min in a helium flux of 100 ml/min.

Malvern Nano ZS light scattering apparatus (Malvern Panalytica, UK) was used for dynamic light scattering (DLS) and ζ-potential measurements of the IONPs. The samples were illuminated by a 633 nm laser, and the intensity of light scattered at an angle of 173^ο^ was measured by an avalanche photodiode. The hydrodynamic mean size diameter (d_z_) and polidyspersity index (PDI) of the samples were calculated using the software provided by the manufacturer. The ζ-potential of the nanoparticles was measured using the technique of laser Doppler velocimetry (LDV).

### Cell cultures

Human glioblastoma multiforme T98G (ATCC, CRL-1690), U87MG (ATCC, HTB-14) and KJT23I (patient-derived) cells as well as human embryonic kidney HEK293T cells (ATCC, CRL-3216), primary human bronchial fibroblasts NHLF and mouse macrophages MAC (ATCC, CCL-46) were routinely cultivated in standard conditions with DMEM medium supplemented with 10% fetal bovine serum (FBS) and 1% antibiotics: penicillin/streptomycin cocktail as it was described elsewhere^33^. For each experiment, cells were harvested with 0.25% Trypsin–EDTA solution (Gibco), counted in a Z2 particle counter (Beckman Coulter), and seeded at an appropriate density into tissue culture plates (Eppendorf).

### Cell proliferation and cytotoxicity tests

Cells were seeded at the density of 2 × 10^4^/cm^2^ in 12-well plates and cultivated for 24 h before the treatment with the culture medium containing IONPs. After 24 and 72 h of cultivation with NPs, the cells were harvested and counted in a Coulter Z2 Counter.

The viability of the cells was estimated with a trypan blue (Sigma-Aldrich, T8154) assay using a Brüker hemocytometer. Cells were seeded into 12-well plates at the density of 2 × 10^4^/cm^2^ and, after 24 or 72 h incubation with IONPs, passaged, centrifuged, resuspended in PBS and mixed with 0.4% dye in ratio 1:1. The cells were quantitatively evaluated after 2 min of incubation.

MTT assay was applied to estimate NPs induced changes in cellular metabolic activity. Cells were seeded into 96-well plates at the density of 5 × 10^3^/cm^2^ and treated with IONPs for 24 or 72 h. Afterwards, 12 µl of thiazolyl blue tetrazolium bromide (Sigma-Aldrich, M5655) solution was added to each well to a final concentration of 0.5 mg/ml and incubated at 37 °C for 4 h. The formed formazan crystals were dissolved with DMSO and the absorbance of the solution was measured using a Multiskan FC Microplate Reader (Thermo Fisher Scientific) at the wavelength of 570 nm.

### Oxidative stress measurements

Cells were seeded on 24-well fluorescence imaging plates and after 24 h of cultivation in standard conditions, treated with medium containing IONPs for 24 h. To determine mitochondrial ROS levels, the cells were incubated with 5 µM of CellROX Orange reagent (Invitrogen, 35050061) for 30 min. Subsequently, the medium was replaced by FluoroBrite DMEM (Invitrogen, A1896702), supplemented with 10% FBS and 1% GlutaMax (Invitrogen, 35050061) and examined with Leica DMI6000B fluorescence microscope equipped with a temperature/CO_2_ chamber. Fluorimetric analysis was performed in ImageJ software^34,35^.

### Cell migration

For migration analysis, the cells were seeded into 12-well plates at the density of 2 × 10^4^/cm^2^, incubated for 24 h and then treated with medium containing IONPs. The movement of cells was recorded for 8 h at 5 min time intervals using a Leica DMI6000B microscope equipped with an interference modulation contrast (IMC), digital DFC360FX camera, CO_2_ chamber (5% CO_2_) and temperature monitoring system (37 °C). The trajectories of individual cells and average values of cell motility parameters, namely speed (µm/min), distance (µm) and displacement (µm) were calculated with Hiro software written by W. Czapla as described in the paper Ryszawy et al*.*^36^.

### Immunofluorescence staining of actin and vinculin

To identify the location of cytoskeletal proteins i.e. F-actin and vinculin, the cells were seeded at the density of 2 × 10^4^/cm^2^ on glass coverslips and incubated for 24 h before administration of medium with IONPs. After 24 and 72 h of cultivation with NPs, cells were fixed in 3.7% formaldehyde for 20 min at 37 °C, permeabilized with 0.1% Triton X-100 for 5 min at 37 °C and incubated in 3% BSA in PBS for 1 h at room temperature. After blocking non-specific antibody binding sites, the samples were incubated in the presence of primary antibody—monoclonal mouse anti-vinculin IgG (Sigma, St. Louis, MO, V9131) for 1 h at room temperature. Then cells were washed and incubated with secondary antibody—Alexa Fluor 488 conjugated donkey anti-mouse IgG (Invitrogen, A21208) for 45 min at room temperature. Visualization of F-actin filaments was carried out by counterstaining with Alexa Fluor 546-phalloidin (Invitrogen, A22283). DNA was stained with Hoechst 33258 (Sigma, St. Louis, MO) at the concentration of 1 µg/ml. Immunostained samples were examined with a Leica DMI6000B microscope equipped with a differential interference contrast (DIC) and total internal reflection fluorescence (TIRF) modules.

### Statistical analysis

Nonparametric Mann–Whitney *U*-test was used to evaluate statistical significance of differences in levels of proliferation, cytotoxicity and ROS obtained for cells treated with IONPs solutions and appropriate controls. As parameters describing cell migration were normally distributed, parametric Student’s *t*-test was applied for their statistical evaluations. In both cases, the differences between cells treated with IONPs and controls were analyzed at the significance level of 5%. The statistical analysis was done with STATISTICA software (version 7.1).

## Supplementary Information

Supplementary Figures.
