# Supplementary material for: The influence of IONPs core size on their biocompatibility and activity in in vitro cellular models
Source: Sci Rep. 2021 Nov 8;11:21808. doi: 10.1038/s41598-021-01237-y (PMC8576000; doi:10.1038/s41598-021-01237-y)
Supplement: Supplementary file 1 — Supplementary Figures. [file 41598_2021_1237_MOESM1_ESM.docx]

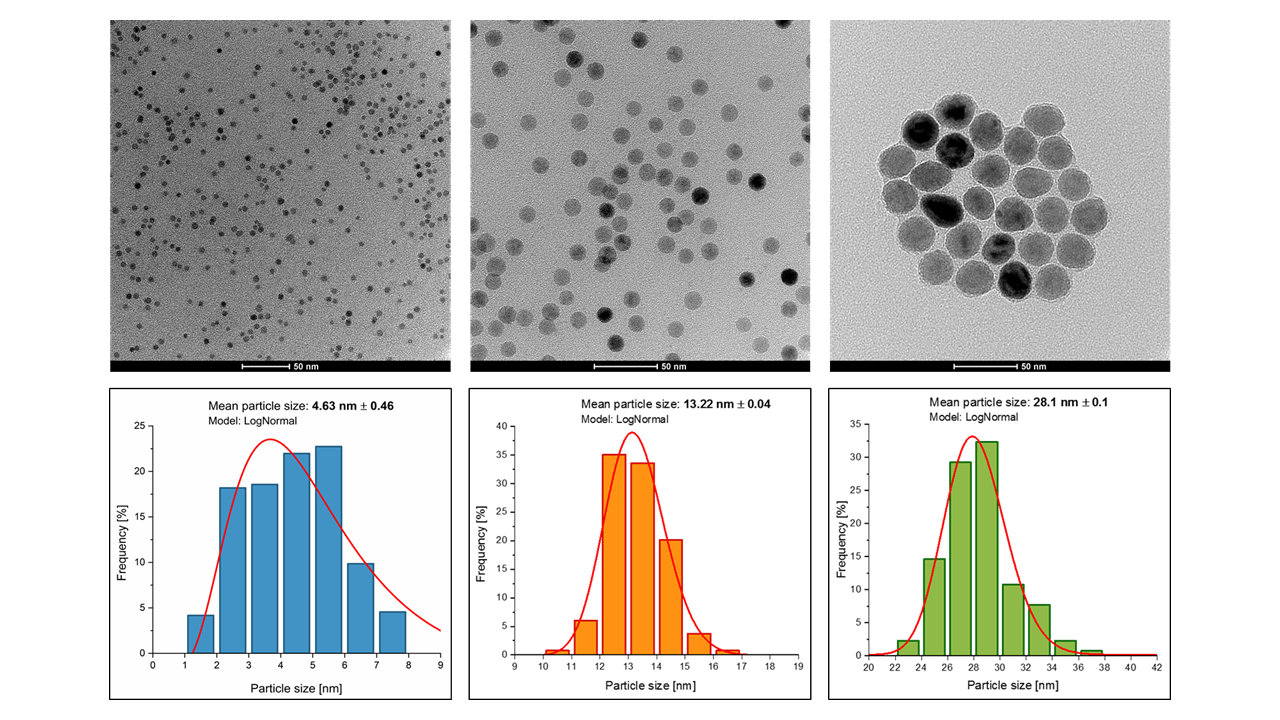


Figure S1 The bright field (BF) TEM images of the nanoparticles used in the study along with the respective histograms of the particle core size distributions and their lognormal fits.


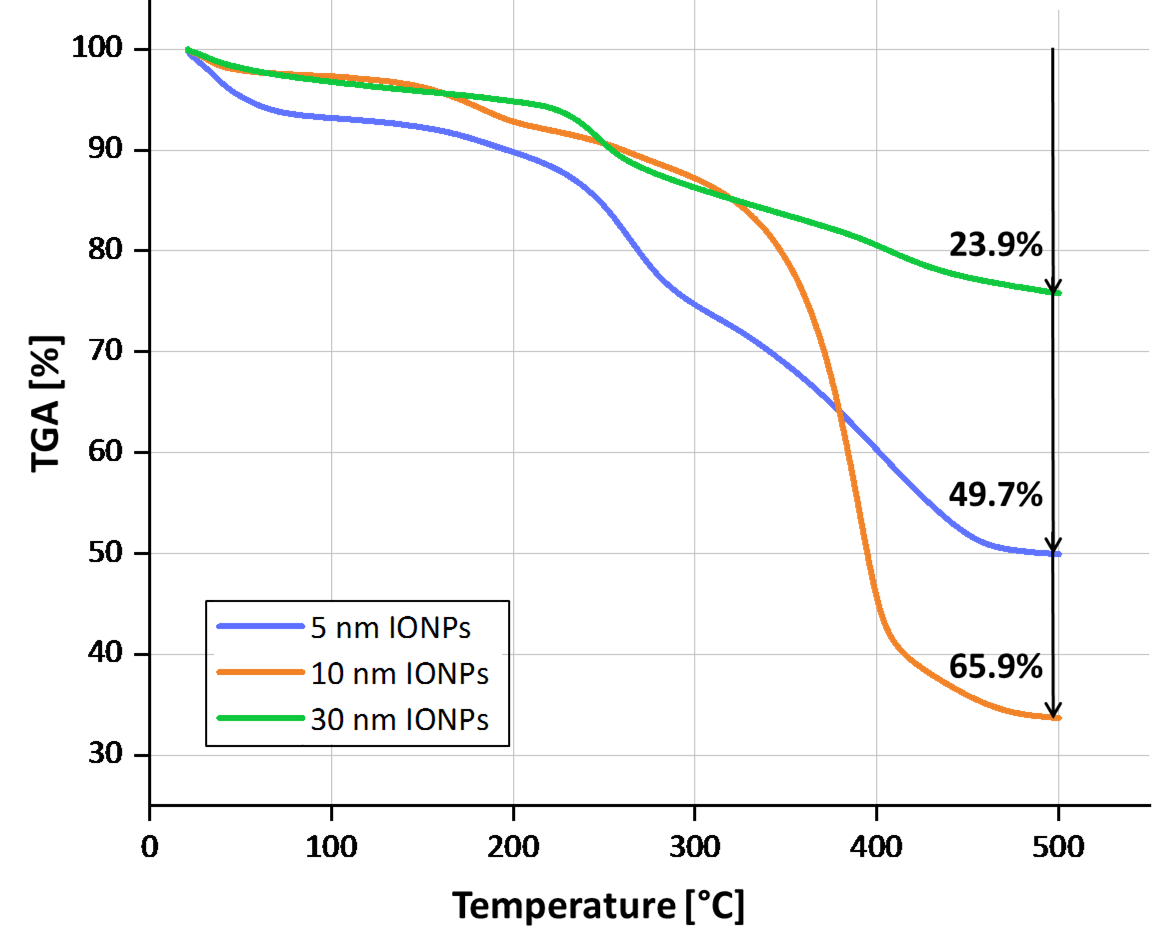


Figure S2 TG results for examined PEGylated iron oxide nanoparticles with the core diameters of 5, 10 and 30 nm.

Figure S3 Dot-plots depict displacement and total length of trajectory (distance) calculated for single HEK293T cells exposed to
5 nm, 10 nm and 30 nm IONPs as well as for corresponding control groups N. Circular plots present trajectories of individual cells.

**HEK293T 5 nm IONPs**

24 h

72 h

N

5 µg Fe/ml

25 µg Fe/ml


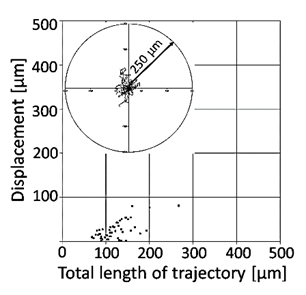

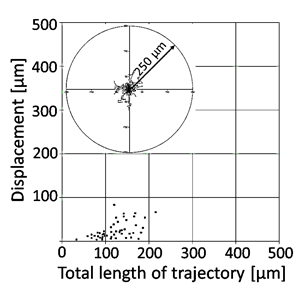

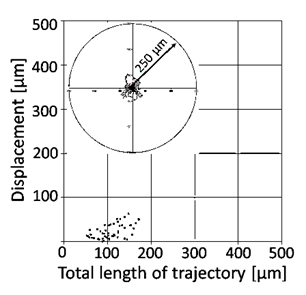

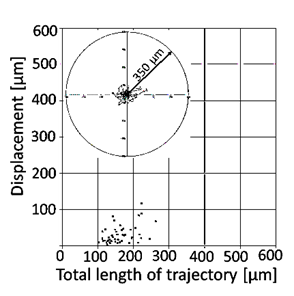

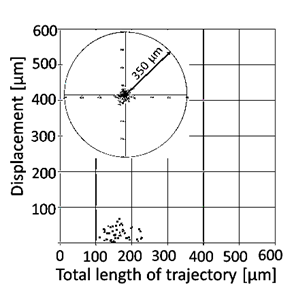

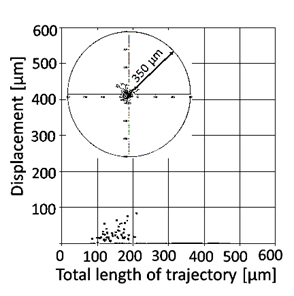


**HEK293T 10 nm IONPs**

24 h

72 h

N

5 µg Fe/ml

25 µg Fe/ml


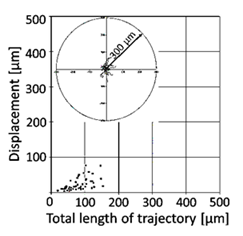

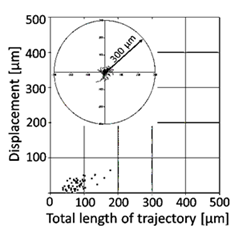

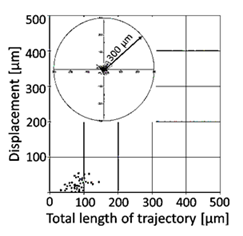

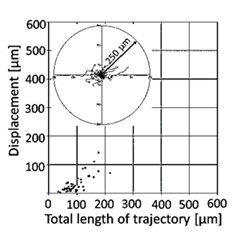

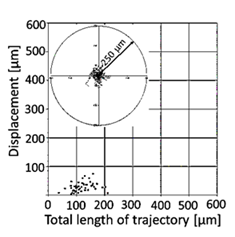

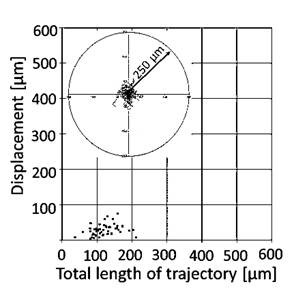


**HEK293T 30 nm IONPs**

24 h

72 h

N

5 µg Fe/ml

25 µg Fe/ml


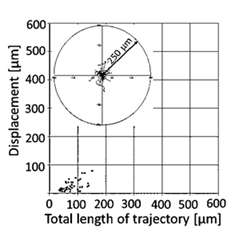

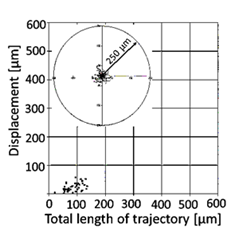

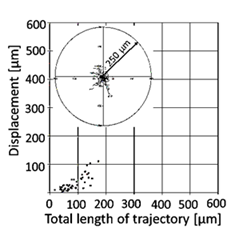

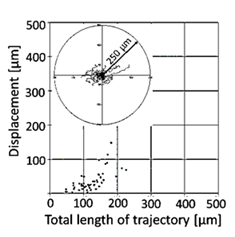

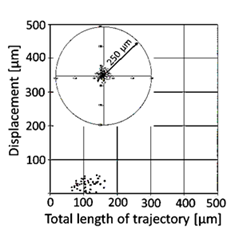

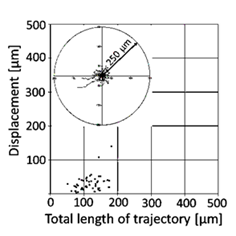


**MACROPHAGES 30 nm IONPs**

24 h

72 h

N

5 µg Fe/ml

25 µg Fe/ml


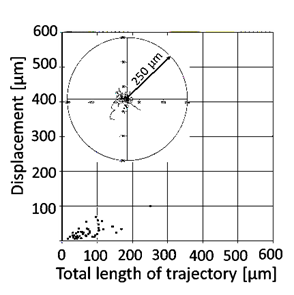

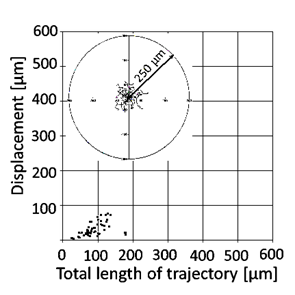

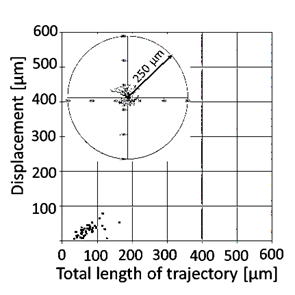

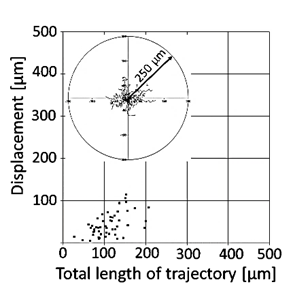

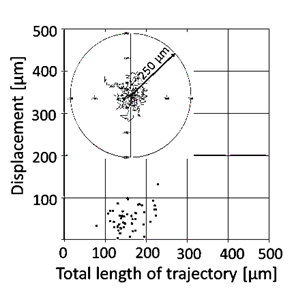

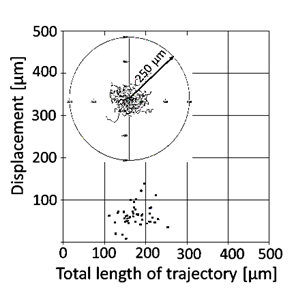


Figure S4 Dot-plots depict displacement and total length of trajectory (distance) calculated for single macrophages exposed to
5 nm, 10 nm and 30 nm IONPs as well as for corresponding control groups N. Circular plots present trajectories of individual cells.

**MACROPHAGES 10 nm IONPs**

24 h

72 h

N

5 µg Fe/ml

25 µg Fe/ml


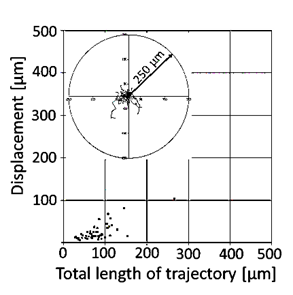

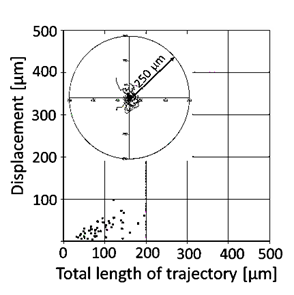

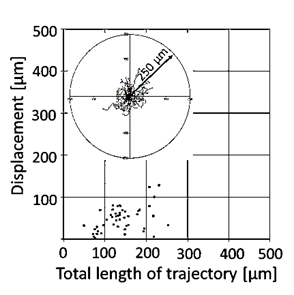

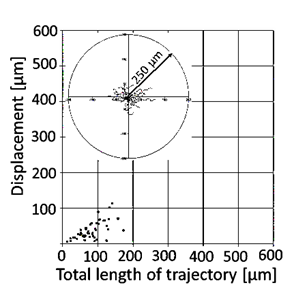

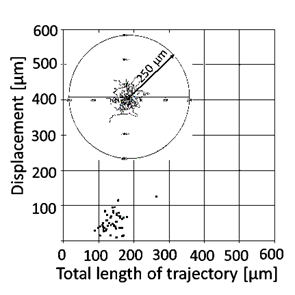

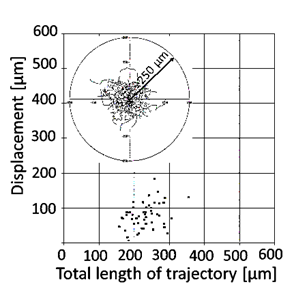


**MACROPHAGES 5 nm IONPs**

24 h

72 h

N

5 µg Fe/ml

25 µg Fe/ml


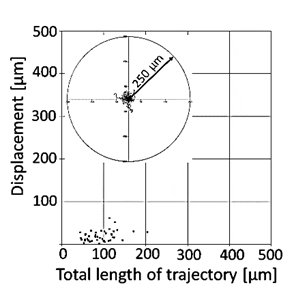

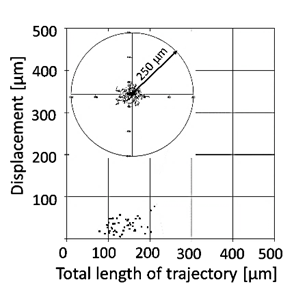

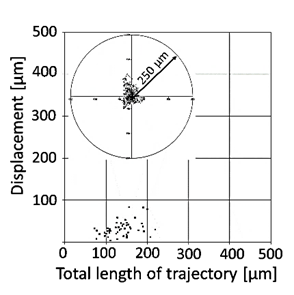

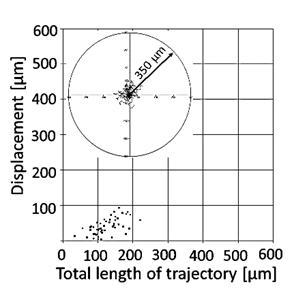

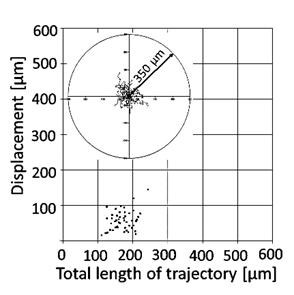

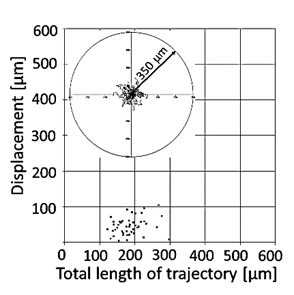


**NHLF 5 nm IONPs**

24 h

72 h

N

5 µg Fe/ml

25 µg Fe/ml


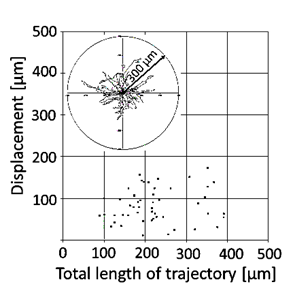

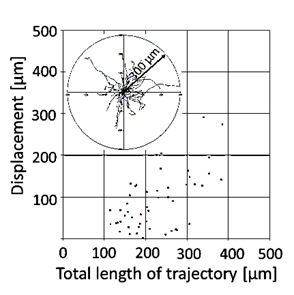

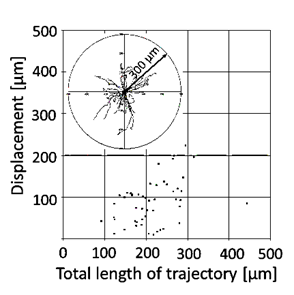

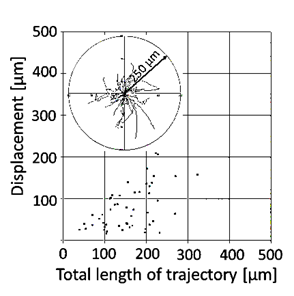

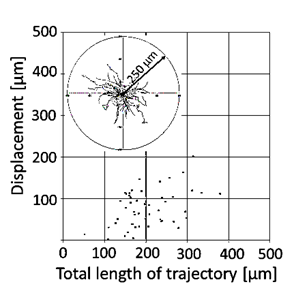

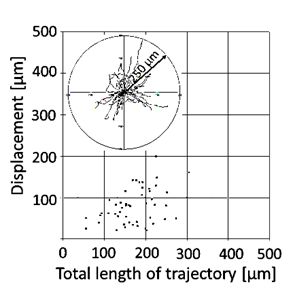


**NHLF 10 nm IONPs**

24 h

72 h

N

5 µg Fe/ml

25 µg Fe/ml


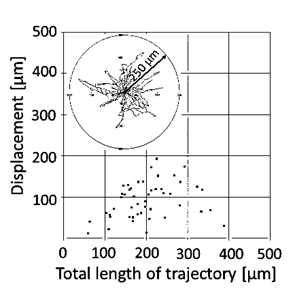

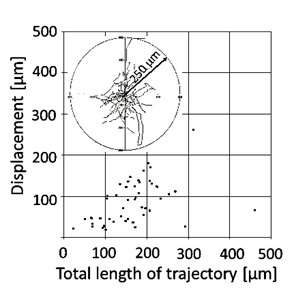

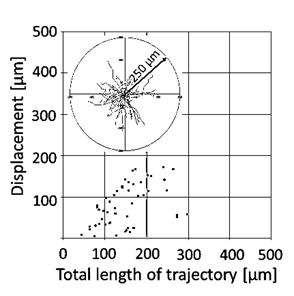

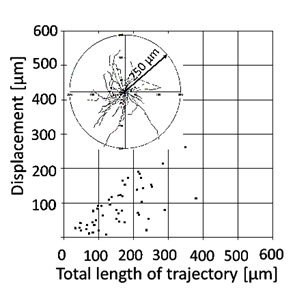

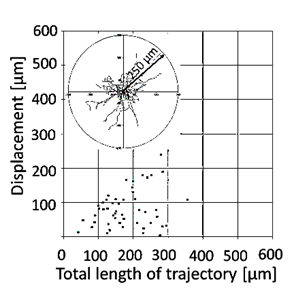

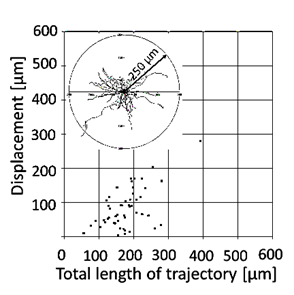


**NHLF 30 nm IONPs**

24 h

72 h

N

5 µg Fe/ml

25 µg Fe/ml


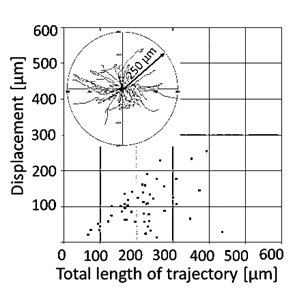

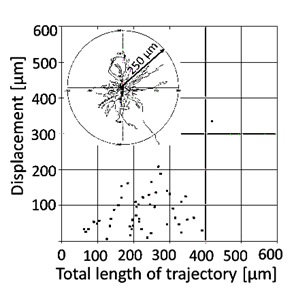

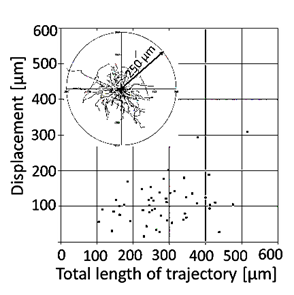

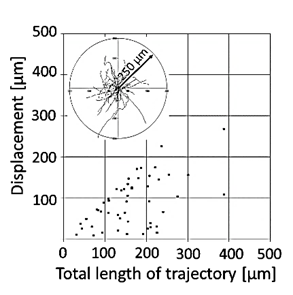

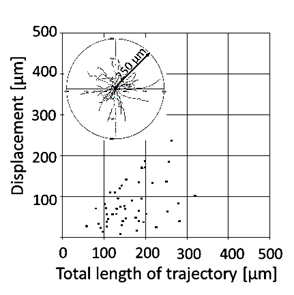

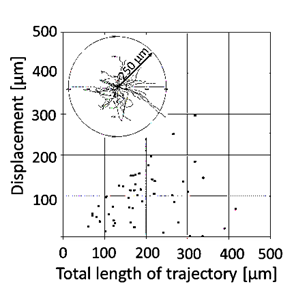


Figure S5 Dot-plots depict displacement and total length of trajectory (distance) calculated for single NHLF cells exposed to 5 nm, 10 nm and 30 nm IONPs as well as for corresponding control groups N. Circular plots present trajectories of individual cells.

**U87 5 nm IONPs**

24 h

72 h

N

5 µg Fe/ml

25 µg Fe/ml


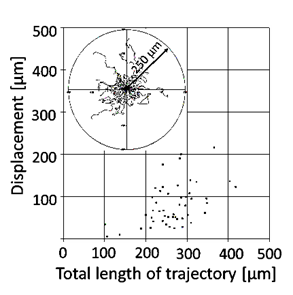

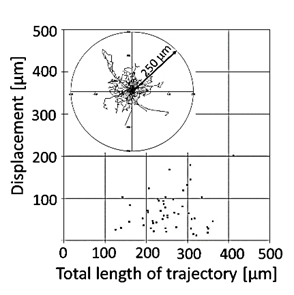

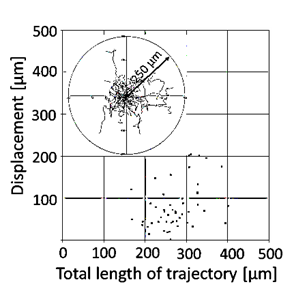

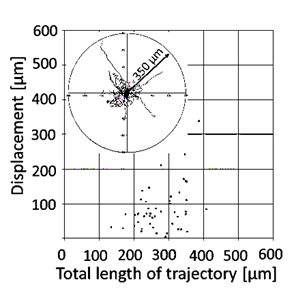

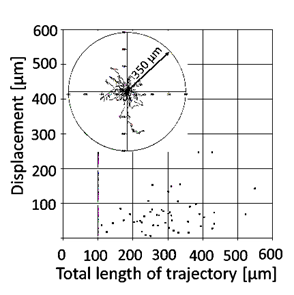

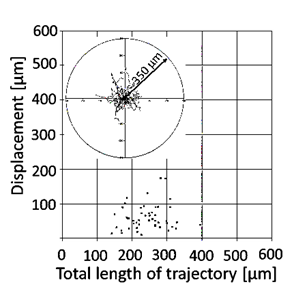


**U87 10 nm IONPs**

24 h

72 h

N

5 µg Fe/ml

25 µg Fe/ml


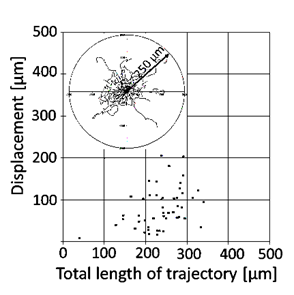

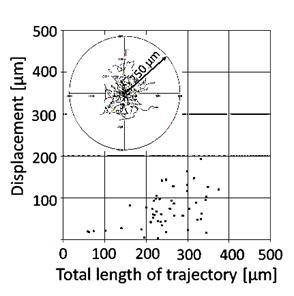

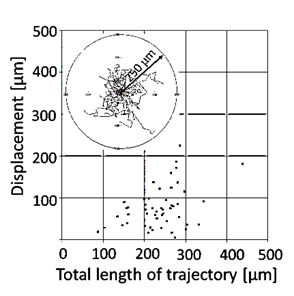

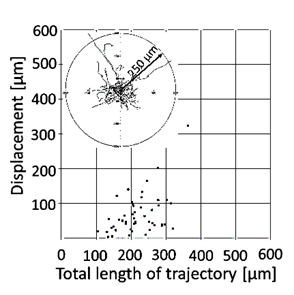

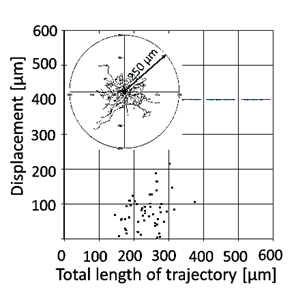

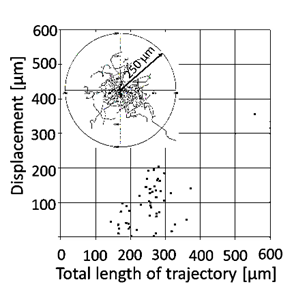


**U87 30 nm IONPs**

24 h

72 h

N

5 µg Fe/ml

25 µg Fe/ml


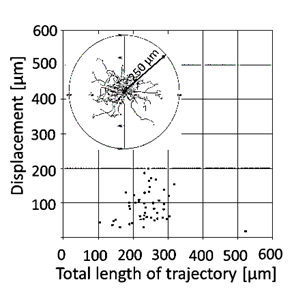

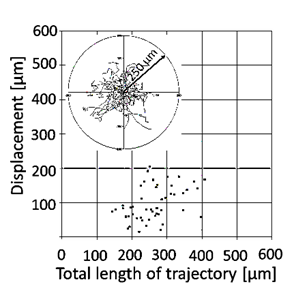

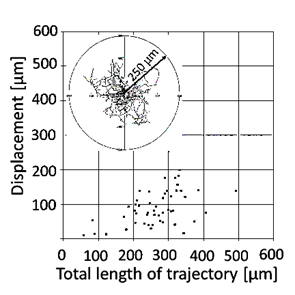

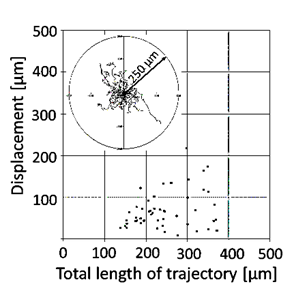

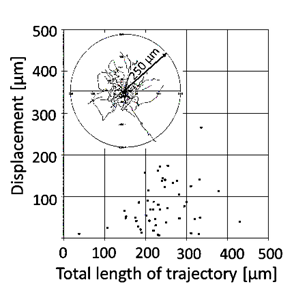

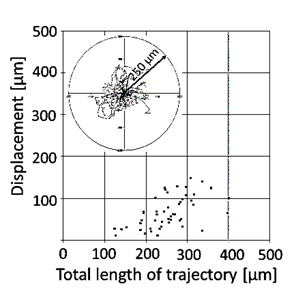


Figure S6 Dot-plots depict displacement and total length of trajectory (distance) calculated for single U87 cells exposed to 5 nm, 10 nm and 30 nm IONPs as well as for corresponding control groups N. Circular plots present trajectories of individual cells.

**T98G 5 nm IONPs**

24 h

72 h

N

5 µg Fe/ml

25 µg Fe/ml


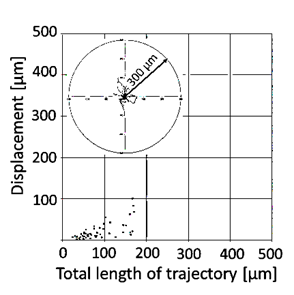

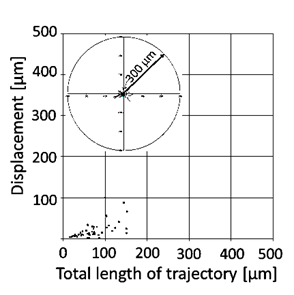

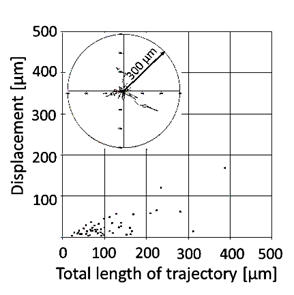

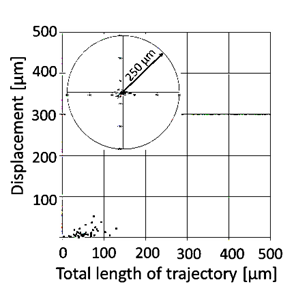

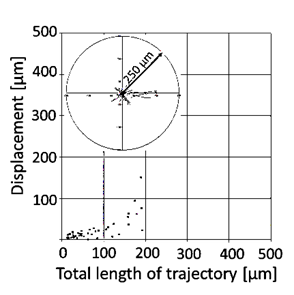

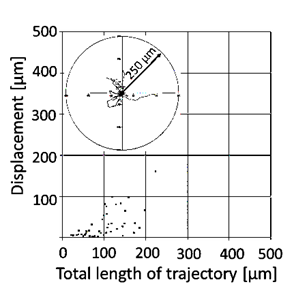


**T98G 10 nm IONPs**

24 h

72 h

N

5 µg Fe/ml

25 µg Fe/ml


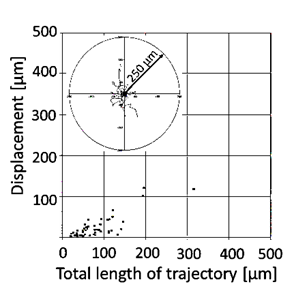

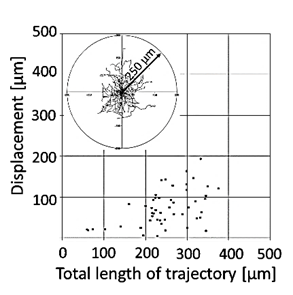

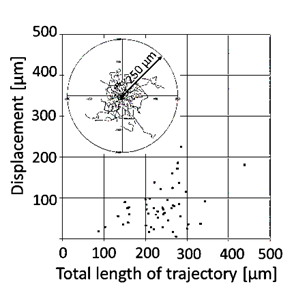

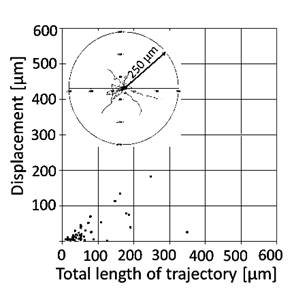

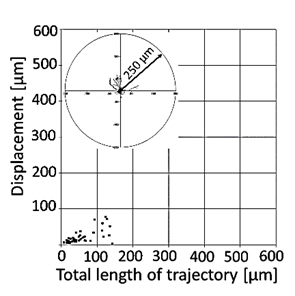

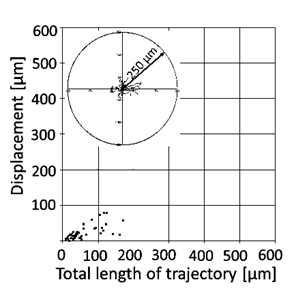


**T98G 30 nm IONPs**

24 h

72 h

N

5 µg Fe/ml

25 µg Fe/ml


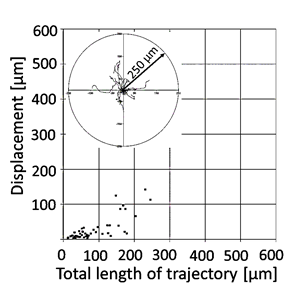

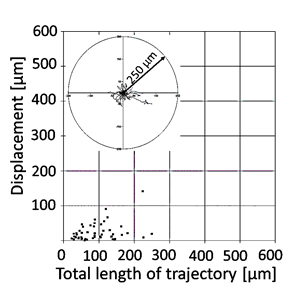

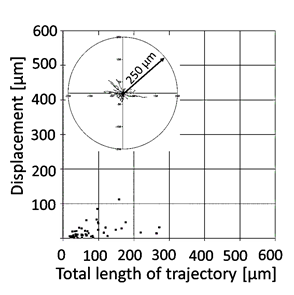

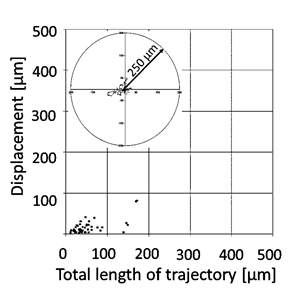

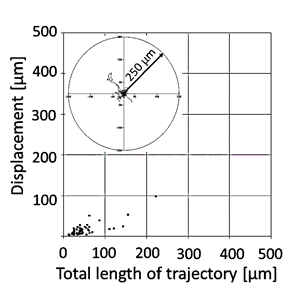

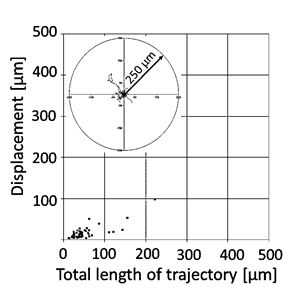


Figure S7 Dot-plots depict displacement and total length of trajectory (distance) calculated for single T98G cells exposed to 5 nm, 10 nm and 30 nm IONPs as well as for corresponding control groups N. Circular plots present trajectories of individual cells.

**KJT23I 5 nm IONPs**

24 h

72 h

N

5 µg Fe/ml

25 µg Fe/ml


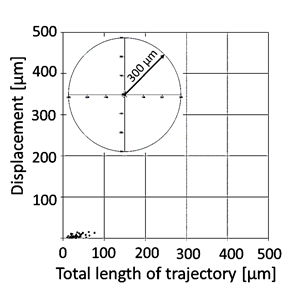

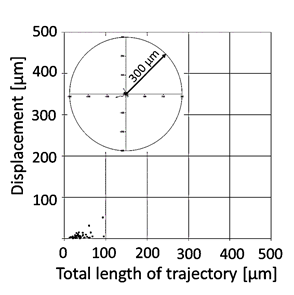

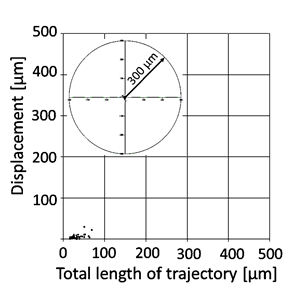

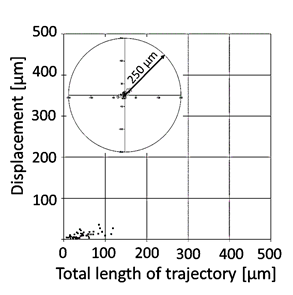

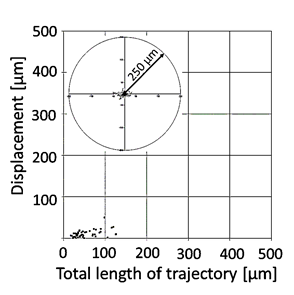

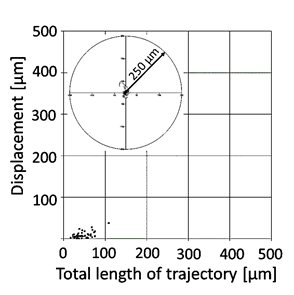


**KJT23I 10 nm IONPs**

24 h

72 h

N

5 µg Fe/ml

25 µg Fe/ml


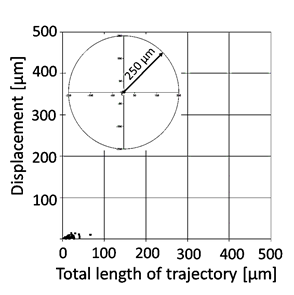

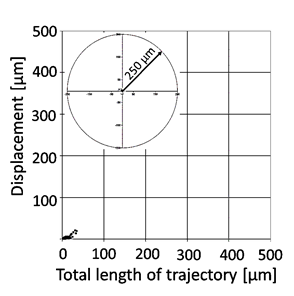


**KJT23I 30 nm IONPs**

24 h

72 h

N

5 µg Fe/ml

25 µg Fe/ml

Figure S8 Dot-plots depict displacement and total length of trajectory (distance) calculated for single KJT23I cells exposed to
5 nm, 10 nm and 30 nm IONPs as well as for corresponding control groups N. Circular plots present trajectories of individual cells.

Figure S9 Cytoskeleton architecture of NHLF cells after 24 and 72-hour long of exposure to IONPs with 5 nm, 10 nm and 30 nm core diameters in concentrations of 5 µg Fe/ml and 25 µg Fe/ml compared with control groups (N). The F-actin filaments are stained on red colour, vinculin on green and DNA on blue.

Figure S10 Cytoskeleton architecture of HEK293T cells after 24 and 72-hour long of exposure to IONPs with 5 nm, 10 nm and 30 nm core diameters in concentrations of 5 µg Fe/ml and 25 µg Fe/ml compared with control groups (N). The F-actin filaments are stained on red colour, vinculin on green and DNA on blue.

Figure S11 Cytoskeleton architecture of T98G cells after 24 and 72-hour long of exposure to IONPs with 5 nm, 10 nm and 30 nm core diameters in concentrations of 5 µg Fe/ml and 25 µg Fe/ml compared with control groups (N). The F-actin filaments are stained on red colour, vinculin on green and DNA on blue.

Figure S12 Cytoskeleton architecture of KJT23I cells after 24 and 72-hour long of exposure to IONPs with 5 nm, 10 nm and 30 nm core diameters in concentrations of 5 µg Fe/ml and 25 µg Fe/ml compared with control groups (N). The F-actin filaments are stained on red colour, vinculin on green and DNA on blue.
